# Cooperative participation of CagA and NFATc1 in the pathogenesis of antibiotics-responsive gastric MALT lymphoma

**DOI:** 10.1186/s12935-024-03552-6

**Published:** 2024-11-18

**Authors:** Hui-Jen Tsai, Kun-Huei Yeh, Chung-Wu Lin, Ming-Shiang Wu, Jyh-Ming Liou, Ping-Ning Hsu, Yi-Shin Zeng, Ming-Feng Wei, Chia-Tung Shun, Hsiu-Po Wang, Li-Tzong Chen, Ann-Lii Cheng, Sung-Hsin Kuo

**Affiliations:** 1 National Institute of Cancer Research, National Health Research Institutes, Tainan, Taiwan; 2 Department of Internal Medicine, Kaohsiung Medical University Hospital, Kaohsiung Medical University, Kaohsiung, Taiwan; 3 Department of Oncology, National Cheng-Kung University Hospital, College of Medicine, National Cheng Kung University, Tainan, Taiwan; 4 Department of Oncology, National Taiwan University Hospital and National Taiwan University College of Medicine, No. 7, Chung-Shan South Rd, Taipei, Taiwan; 5 Cancer Research Center, National Taiwan University College of Medicine, Taipei, Taiwan; 6 Graduate Institute of Oncology, National Taiwan University College of Medicine, Taipei, Taiwan; 7 Department of Pathology, National Taiwan University Hospital and National Taiwan University College of Medicine, Taipei, Taiwan; 8 Department of Pathology and Laboratory Medicine, Koo Foundation Sun Yat-Sen Cancer Center, Taipei, Taiwan; 9 Department of Internal Medicine, National Taiwan University Hospital and National Taiwan University College of Medicine, Taipei, Taiwan; 10 Department of Internal Medicine, National Taiwan University Cancer Center, National Taiwan University College of Medicine, Taipei, Taiwan; 11 Department of Medical Oncology, National Taiwan University Cancer Center, National Taiwan University College of Medicine, Taipei, Taiwan

**Keywords:** MALT lymphoma, CagA, NFATc1, *Helicobacter pylori*, Stomach

## Abstract

**Background:**

This study aimed to explore whether cytotoxin-associated gene A (CagA) can inhibit cell cycle progression by activating nuclear factor of activated T cells (NFAT) in lymphoma B cells and contribute to *Helicobacter pylori* eradication (HPE) responsiveness (complete remission [CR] after HPE) in gastric mucosa-associated lymphoid tissue (MALT) lymphoma.

**Materials and Methods:**

We co-cultured three B-lymphoma cell lines (MA-1, OCI-Ly3, and OCI-Ly7) with HP strains (derived from HPE-responsive gastric MALT lymphoma) and evaluated the expression patterns of CagA, phosphorylated (p)-CagA (CagA^P−Tyr^), and CagA-signaling molecules, cell-cycle inhibitors, p-NFATc1 (Ser^172^), and NFATc1 using western blotting*.* Furthermore, we evaluated the association between nuclear NFATc1 expression in the tumor cells of 91 patients who received first-line HPE (59 patients with HPE responsiveness and 32 without HPE responsiveness) and HPE responsiveness and CagA expression in tumor cells.

**Results:**

In HP strains co-cultured with B cell lymphoma cell lines, CagA was translocated to the nucleus through tyrosine phosphorylation (CagA^P−Tyr^) and simultaneously dephosphorylated NFATc1, subsequently causing nuclear NFATc1 translocation and stimulating the expression of p-SHP-2/p-ERK/Bcl-xL. Activated NFATc1 causes G1 cell cycle retardation in both MA-1 and OCI-Ly3 cells by triggering p21 and p27 production. Nuclear NFATc1 localization was significantly associated with the presence of CagA in gastric MALT lymphomas (80% [41/51] vs. 33% [13/40]; *p* < 0.001) and with HPE responsiveness (73% [43/59] vs. 25% [8/32]; *p* < 0.001). Patients exhibiting both the presence of CagA and nuclear NFATc1 localization responded more rapidly to HPE than those without (median interval to CR, 4.00 vs. 6.00 months, *p* = 0.003).

**Conclusions:**

Our findings indicated that CagA and NFATc1 cooperatively participate in the lymphomagenesis of HPE-responsive gastric MALT lymphoma.

**Supplementary Information:**

The online version contains supplementary material available at 10.1186/s12935-024-03552-6.

## Introduction

Several studies have demonstrated that patients with localized *Helicobacter pylori* (HP)-positive gastric mucosa-associated lymphoid tissue (MALT) lymphoma (also known as extranodal marginal zone B-cell lymphoma) are highly responsive to first-line antibiotic treatment combined with proton pump inhibitors [1–3]. Previous reports suggest that in the early lymphomagenesis of MALT lymphoma, the proliferative response of lymphoma cells is partially dependent on the help of HP*-*exclusive intratumoral T cells by HP antigens, CD40-CD40 ligand signaling, T helper-2-type cytokines, or communication between co-stimulatory molecules (CD86) and HP-specific T cells [1, 3–7]. In addition to classic T-cell mechanisms, previous studies have shown that self-antigen-stimulating B-cell receptor signaling and CD4^+^CD25^+^Foxp3^+^ regulatory T cell-originated signals participate in the pathogenesis of gastric MALT lymphoma [8–12].

HP-encoded cytotoxin-associated gene A (CagA) protein has been shown to bind to Src homology-2 domain-containing phosphatase (SHP-2) in gastric epithelial cells after tyrosine phosphorylation at its specific glutamic acid-proline-isoleucine-tyrosine-alanine (EPIYA) segments [13–15]. A previous study reported that HP CagA translocates to human B lymphocytes through tyrosine phosphorylation and promotes the proliferation of these cells by triggering SHP-2-related signals, such as extracellular signal-regulated kinase (ERK) and p38 mitogen-activated protein kinase (MAPK) signaling, as well as by activating the production of B-cell lymphoma 2 (Bcl-2) and B-cell lymphoma-extra-large (Bcl-xL) [16]. Furthermore, in lymphoma samples from patients with gastric MALT lymphoma who received first-line HP eradication therapy (HPE), nuclear localization of CagA was significantly correlated with the presence of CagA signaling pathway-associated molecules, including phosphorylated-(p)-SHP-2, p-ERK, p-p38 MAPK, Bcl-2, and Bcl-xL in lymphoma cells [17]. It was also reported that CagA and its subsequent phosphorylation of SHP-2 were involved in the development of HP-related gastrointestinal and hematopoietic neoplasms [18].

In addition to deregulating SHP-2, CagA can impede cell cycle progression by triggering nuclear factor of activated T cells (NFAT) and its regulated genes such as *p21* (a cyclin-dependent kinase [CDK] inhibitor) in gastric epithelial cells [19]*.* The calcium-dependent serine/threonine phosphatase calcineurin, which activates NFAT signaling, plays a crucial role in the pathogenesis of certain B-cell lymphoid neoplasms [20, 21]. Previous studies have revealed nuclear localization of nuclear factor of activated T cell 1 (NFATc1, also known as NFAT2) in lymphoma cells of certain subtypes, including MALT lymphoma, diffuse large B-cell lymphoma (DLBCL), Burkitt’s lymphoma, and Hodgkin’s lymphoma [22, 23].

In this study, we investigated whether CagA from HP translocates to B-lymphocytes cells, subsequently causing nuclear localization of p-CagA (tyrosine phosphorylation of CagA, CagA^P−Tyr^) and activation of CagA-signaling molecules (phosphorylation of SHP-2 and ERK as well as the expression of Bcl-xL) in lymphoma B-cells co-cultured with HP strains. Furthermore, we explored whether the nuclear localization of CagA simultaneously activated the nuclear localization of NFATc1 in HP co-cultured lymphoma B cells through the activation of p21. We also examined the association between nuclear localization of NFATc1 and CagA in lymphoma cells and HPE responsiveness (complete remission [CR] of lymphoma after HPE) in patients with gastric MALT lymphoma who received first-line HPE. Our aim was to clarify whether, in addition to CagA, the nuclear localization of NFATc1 is involved in the pathogenesis of HPE-responsive gastric MALT lymphoma.

## Materials and methods

### Lymphoma cell lines and gastric epithelial cells

The DLBCL cell lines OCI-Ly3, OCI-Ly7, MA-1, and subclones of MA-1 (MA-1#46), and the gastric epithelial cell line AGS were used in this study. AGS cells (American Type Culture Collection [ATCC]^®^ CRL-1739™) were purchased from the ATCC (Manassas, VA, USA). OCI-Ly3 and OCI-Ly7 cells were provided by Dr. Louis M. Staudt (National Institutes of Health, Bethesda, Maryland, USA). OCI-Ly3 is an activated B-cell-like (ABC) DLBCL cell line that contains no translocations of (11;18) (q21;q21) [24]. Our established MA-1 and MA-1#46 cells are both t(14;18) (q32;q21)/IGH-MALT1-harboring lymphoma cells with a distinct morphology and 8-code short tandem repeat (STR) repeats that are identical to Pfeiffer cells, a well-recognized DLBCL cell line [25–27]. Thus, MA-1 cells are considered a derivative of Pfeiffer (Capes-Davis A, PubMed Commons on Tsai et al. [25]). The cellular origin of MALT lymphomas is conventionally considered to be non-germinal central B-like (GCB) cells [1, 28]. In addition to (11;18) (q21;−q21), certain MALT lymphomas harbor a chromosomal translocation of t(14;18)(q32;q21)/IGH-MALT1 [29–31]. Therefore, in this study, we used two B-cell lymphoma cell lines: OCI-Ly3, a non-GCB/ABC subtype of DLBCL, and MA-1 or MA-1#46, a t(14;18)(q32;q21)/IGH-MALT1-positive DLBCL, to mimic the in vitro biology of gastric MALT lymphoma. In addition to OCI-Ly3 and MA-1 cells, we used a GCB-origin DLBCL cell line, OCI-Ly7 [24], and a gastric epithelial cell line, AGS, to assess whether CagA could activate SHP-2-dependent signaling as well as NFATc1 and p21, in B lymphocytes and gastric epithelial cells co-cultured with HP strains.

In November 2016, the following cell lines were sent to the Center for Genomic Medicine at the National Cheng Kung University of Taiwan for genotyping: OCI-Ly3, OCI-Ly7, MA-1, MA-1 #46, and AGS. The genotyping results showed an STR-polymerase chain reaction (PCR) of the DNA profile of these lymphoma cells, similar to those in the National Institutes of Biomedical Innovation, Health, and Nutrition (JCRB) database. The OCI-Ly3, OCI-Ly7, MA-1, and MA-1#46 lymphoma cell lines were cultured in Roswell Park Memorial Institute (RPMI) 1640 medium (HyClone, Logan, UT, USA), and AGS cells were cultured in Ham’s F-12 K medium (Gibco, New York, NY, USA). We supplemented these cell lines using 10% fetal bovine serum (FBS; HyClone, USA) and 1% penicillin/streptomycin (Gibco, USA), and cultured these cells in an incubator in 5% CO_2_ at 37 °C.

### HP strain, and lymphoma cell lines and gastric epithelial cells co-cultured with HP

HP strains HM#2, HM#8, and HM#12 were isolated from patients 2, 8, and 12 with HPE-responsive gastric MALT lymphoma, respectively, whereas HP strain HS235 was isolated from one patient with HP-positive gastritis. These HP strains were grown on blood agar plates under microaerobic conditions for 3 days, after which they were harvested and washed with serum-free and antibiotic-free RPMI 1640 medium. We accumulated and rinsed OCI-Ly3, OCI-Ly7, MA-1, MA-1#46, and AGS cells (3 × 10^6^) twice with phosphate-buffered saline (PBS). Subsequently, the cells were infected with HP at a multiplicity of infection (MOI) of 150 (150 bacteria per cell) and co-cultured with HP for 1.5 h in serum-free and antibiotic-free RPMI 1640 or F-12 K medium.

Subsequently, the infected cells were centrifuged at 1,300 rpm for 5 min and washed once with PBS to remove HP. Washed cells were cultured in RPMI 1640 or F-12 K medium and harvested at the indicated time points. The harvested cells were pelleted by centrifugation (1,300 rpm for 5 min), the cell pellet was rinsed twice with PBS, and the cells were lysed to collect total lysates or nuclear and cytoplasmic protein fractions, as previously described [25, 32].

### Immunoblotting analysis

The detailed methods are described in the Supplementary Materials and Methods [16, 17, 22, 25, 32].

### Cell proliferation assay and cell cycle analysis

The detailed methods are described in the Supplementary Materials and Methods.

### Patients, treatment, and tumor evaluation

Between January 1, 2002, and December 31, 2016, 91 patients with HP-positive stage IE and IIE1 gastric MALT lymphoma who had received first-line HPE and had available lymphoma samples were included in this study. In the current study, the presence of HP infection in patients with gastric MALT lymphoma was defined as a positive result on urease biopsy, histology, serology, or a urease breath test [33, 34]. Histological diagnosis of MALT lymphoma of the stomach was based on the criteria for marginal zone cell lymphoma, as previously described [32, 35]. All patients with gastric MALT lymphoma underwent a standard staging workup, and stages IE and IIE1 were classified according to the Musshoff modification of the Ann Arbor staging system [3, 36]. Tumor regressions (including CR) after HPE were histologically evaluated based on the histological scoring system criteria of the Groupe d’Etude des Lymphomes de l’Adult (GELA) [36, 37]. In the current study, patients whose tumors resolved to CR after successful HPE were considered to have HPE-responsive tumors, whereas those who showed tumor progression at any time during follow-up or did not achieve CR at the end of 24 months after completing successful HPE were considered to have HPE-irresponsive tumors [32, 36, 37].

### Immunohistochemistry, immunohistochemical scoring, and confocal laser-scanning microscopy (CLSM)

Immunohistochemical analysis of CagA (dilution 1:50; A10; sc-28368, Santa Cruz Biotechnology) and NFATc1 (dilution 1:50; 7A6, sc-7294; Santa Cruz Biotechnology) was performed on paraffin-embedded sections of pretreatment endoscopic biopsy specimens [17, 22]. The expression patterns of CagA and NFATc1 in the lymphoma cells were visualized using an indirect immunoperoxidase assay. In the current study, we used cell blocks from the CagA-translocated human B-cell line (in which nuclear localization of NFATc1 was confirmed) as positive controls for the staining of CagA and NFATc1.

We considered CagA to be present in lymphoma cells if moderate or strong immunostaining of CagA (appreciable brown staining distinctly marking the nucleus or cytoplasm of lymphoma cells) was expressed in ≥ 10% of lymphoma cells [38]. The percentage of positive nuclear localization of NFATc1 in the lymphoma cells was averaged to obtain an immunohistological score ranging from 0 to 100%. We defined positive nuclear localization of NFATc1 based on the following criteria: moderate or strong nuclear immunostaining of 10–30% (staining intensity score = 2) or > 30% of the nuclear cells were stained (staining intensity score = 3), whereas the absence of nuclear localization of NFATc1 was defined as the absence of nuclear localization of NFATc1 (staining intensity score = 0) or positive nuclear localization of NFATc1 was detected in < 10% of lymphoma cells (staining intensity score = 1).

A laser scanning microscope (TCS SP5, Leica Microsystems, Wetzlar, Germany) was used to detect whether CagA and NFATc1 co-localized in the nuclei of lymphoma cells. We used the fluorescence of fluorescein isothiocyanate-CagA (green), rhodamine-NFATc1 (red), and 4’,6-diamidino-2-phenylindole (DAPI; detection of nuclei; blue, Biotium, 40,043) as primary antibodies, and fluorescein isothiocyanate-labeled donkey anti-mouse IgG or rhodamine-labeled goat anti-rabbit IgG as secondary antibodies.

### Assessment of the t (11;18)(q21;q21) in lymphoma cells of patients with gastric MALT lymphoma

The detailed methods are described in the Supplementary Materials and Methods [1, 3, 8, 31, 39, 40].

### Ethics

The Institutional Review Board of the Research Ethics Committee of National Taiwan University Hospital (approval number: 201801030RINC) approved the study protocol, which included clinical data collection, pathological review, and molecular studies.

### Statistical analysis

The results of immunoblotting are presented as mean ± standard error (SE), and statistical significance was determined using one-way analysis of variance (ANOVA). We used the chi-squared test, Fisher’s exact test, Student’s t-test, and one-way ANOVA to compare the clinical characteristics, presence of CagA, nuclear localization of NFATc1 in the HPE-responsive (CR of lymphoma after HPE) and HPE-irresponsive (non-CR of lymphoma after HPE) subgroups, and clinicopathological features between the presence of nuclear localization of NFATc1 and the absence of nuclear localization of NFATc1 subgroups. Analyses were conducted using the follow-up data available on December 31, 2019. We used a logistic regression model for multivariate analyses of potential factors associated with HPE responsiveness (factors with *p* < 0.1) from the univariate analyses. Kaplan–Meier analysis was used to calculate the time to first CR in patients with HPE responsiveness after completing HPE. Statistical significance was set at *p* < 0.05 (two-sided) in the comparative tests.

## Results

### HP infection induces CagA translocation and CagA tyrosine-phosphorylation and simultaneously causes nuclear NFATc1 localization in lymphoma B-cells and gastric epithelial cell lines

Previous studies have demonstrated that the nuclear translocation of NFATc1 is triggered by the inflow of extracellular calcium and its downstream molecule, calcineurin, and the subsequent dephosphorylation of NFATc1 by activated calcineurin [20, 21, 41]. In this study, we aimed to explore whether HP CagA can induce the nuclear localization of NFATc1 through CagA tyrosine phosphorylation and NFATc1 dephosphorylation in HP co-cultured lymphoma B-cell lines.

**Fig. 1:**
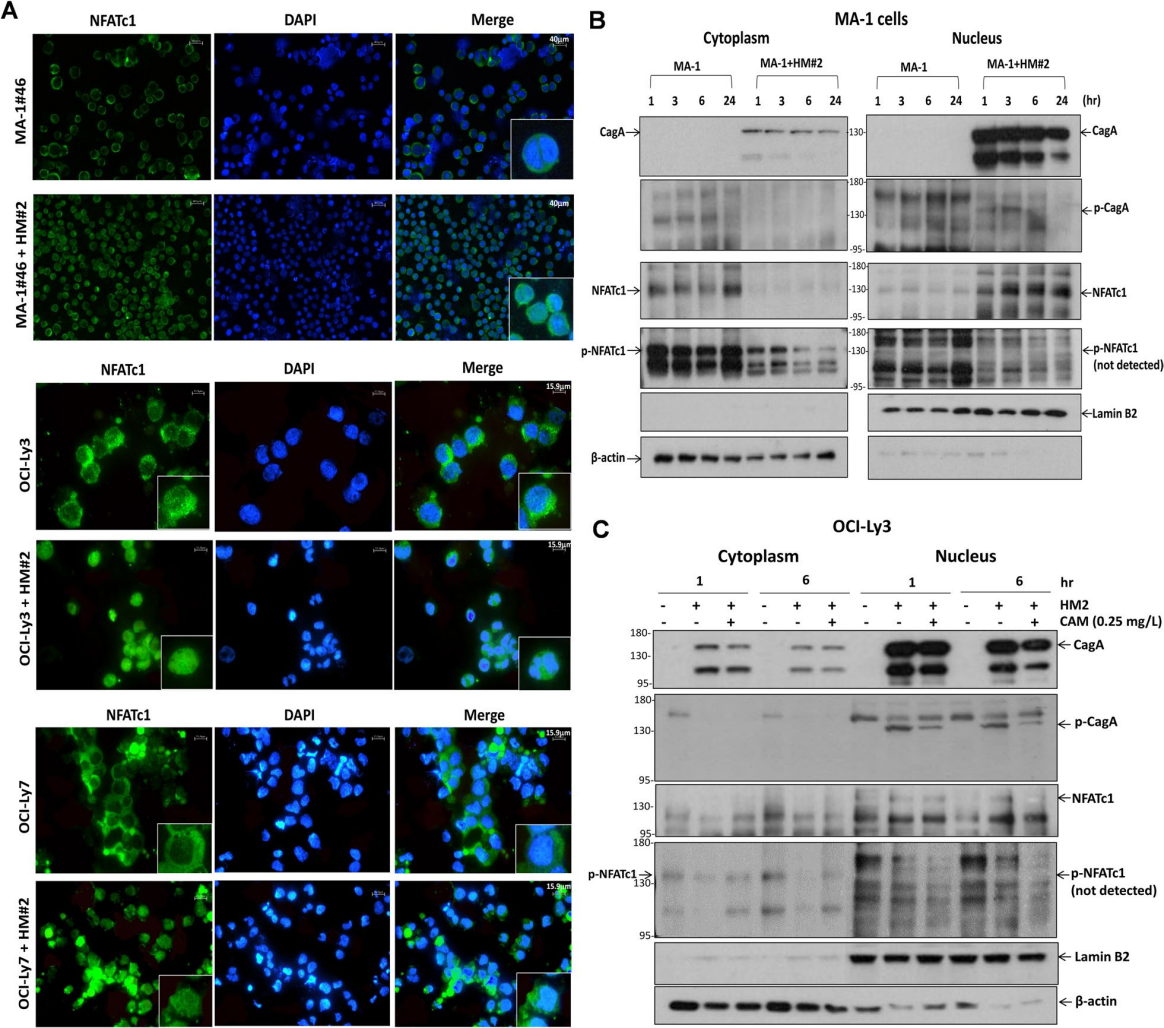
CagA, tyrosine-phosphorylated CagA, and NFATc1 can be translocated to the nucleus in HP-co-cultured DLBCL cells. **A** Immunofluorescence showed that NFATc1 translocated to the nucleus in HP (HM#2)-co-cultured MA-1#46 cells (upper panel) (scale bar = 40 μm). Similarly, NFATc1 translocated to the nucleus in HP (HM#2)-co-cultured OCI-Ly3 cells (ABC-DLBCL) (middle panel) (scale bar = 15.9 μm). In HP (HM#2)-co-cultured OCI-Ly7 cells (GCB-DLBCL) (lower panel), NFATc1 also translocated to the nucleus (scale bar = 15.9 μm). **B** In HP (HM#2)-co-cultured MA-1 cells, nuclear CagA expression was observed at 1, 3, 6, and 24 h, whereas phosphorylated (p)-CagA (CagA^P−Tyr^) expression was observed at 1 and 3 h after HP stimulation. However, the effect decreased at 6 h and eventually disappeared at 24 h. Simultaneously, nuclear NFATc1 expression was observed at 1, 3, and 6 h after HP stimulation and continued until 24 h in HP (HM#2)-co-cultured MA-1 cells. Compared with that noted in the control MA-1 cells, the expression of cytoplasmic p-NFATc1 decreased at 1 and 3 h, and the decreased effect was significantly noted at 6 and 24 h in HP (HM#2)-co-cultured MA-1 cells. The p-NFATc1 expression was not detected in the nucleus of MA-1 cells. **C** CagA and p-CagA translocated to the nucleus of the HP (HM#2)-co-cultured OCI-Ly3 cells at 1 and 6 h. Simultaneously, nuclear expression of NFATc1 was detected at 1 and 6 h after HP stimulation. In addition, the cytoplasmic expression of p-NFATc1 was significantly diminished at 1 and 6 h in HP (HM#2)-co-cultured OCI-Ly3 cells. After the treatment of clarithromycin (CAM, 0.25 mg/L) in HP (HM#2)-co-cultured OCI-Ly3 cells, CagA and p-CagA expression decreased at 6 h, and nuclear NFATc1 expression decreased simultaneously at 6 h, whereas cytoplasmic expression of p-NFATc1 was reversed at 1 and 6 h

The B-cell lymphoma cell lines MA-1, MA-1#46, OCI-Ly3, and OCI-Ly7 were infected with or without the HP strain HM#2 for 2 h and washed to remove HP for further culture. HP-infected MA-1, MA-1#46, OCI-Ly3, and OCI-Ly7 cells were cultured and collected at indicated time points for up to 24 h (Supplementary Fig. S1A). Immunofluorescence data (26 h after initiating coculture with or without the HP strain) showed that NFATc1 was localized in the nucleus of MA-1#46, OCI-Ly3, and OCI-Ly7 cells after infection with HP (HM#2) (Fig. 1A). Nuclear translocation of CagA was detected in MA-1 cells at 1, 3, and 6 h, and the intensity of the blotting decreased slightly 24 h after HP (HM#2) infection (Fig. 1B). The expression of nuclear p-CagA (CagA^P−Tyr^) was observed at 1 and 3 h after HP stimulation; however, the effect decreased at 6 h and eventually disappeared at 24 h. In addition, nuclear localization of NFATc1 was observed at the same time points and for up to 24 h in MA-1 cells co-cultured with HP (HM#2). Decreased cytoplasmic expression of p-NFATc1 (Ser^172^) was observed at 1 and 3 h after HP stimulation, and this effect became predominant at 6 and 24 h after HP stimulation, compared with that noted in non-HP co-cultured MA-1 cells (Fig. 1B). As shown in Supplementary Fig. S1B, in HP (HM#12)-co-cultured MA-1 cells, CagA-induced nuclear NFATc1 translocation was abolished by inhibiting calcineurin with cyclosporine A (CsA), whereas CagA was unaffected. These findings indicated that NFATc1 is activated by the CagA-dependent calcineurin signaling pathway.

As shown in Supplementary Fig. S1B, the expression of nuclear HP vacuolating cytotoxin A (VacA) was not obvious in HP-co-cultured MA-1 cells compared with that in HP-uninfected MA-1 cells. In addition, the administration of CsA did not alter the expression of VacA. These findings suggest that VacA plays a lesser role in HP-related lymphomagenesis of lymphoma B cells.

In OCI-Ly3 (ABC-origin DLBCL) cells, nuclear translocation of CagA, nuclear p-CagA expression, and nuclear localization of NFATc1 were simultaneously observed 1 and 6 h after HP (HM#2) infection, whereas cytoplasmic p-NFATc1 expression decreased at 1 and 6 h after HP (HM#2) infection (Fig. 1C). Considering that clarithromycin is the primary drug used to eradicate HP, we used 1 mg/L, 0.5 mg/L, 0.25 mg/L, 0.125 mg/L, and 0 mg/L of clarithromycin to treat HP on the culture plate. As shown in Supplementary Fig. S2A, the inhibitory effect on the growth of HP strain (HM#2) was dose-dependent, and 0.25 mg/L of clarithromycin effectively inhibited the growth of HP strain (HM#2). In the following experiment, HP (HM#2)-co-cultured OCI-Ly3 cells were treated with clarithromycin (0.25 mg/L) to assess whether abolishing HP stimulation can inhibit the expression of CagA and its tyrosine phosphorylation. We found that clarithromycin simultaneously decreased the expression of nuclear CagA and p-CagA and the nuclear localization of NFATc1 after 6 h, whereas cytoplasmic p-NFATc1 expression was reversed after clarithromycin treatment (Fig. 1C).

**Fig. 2:**
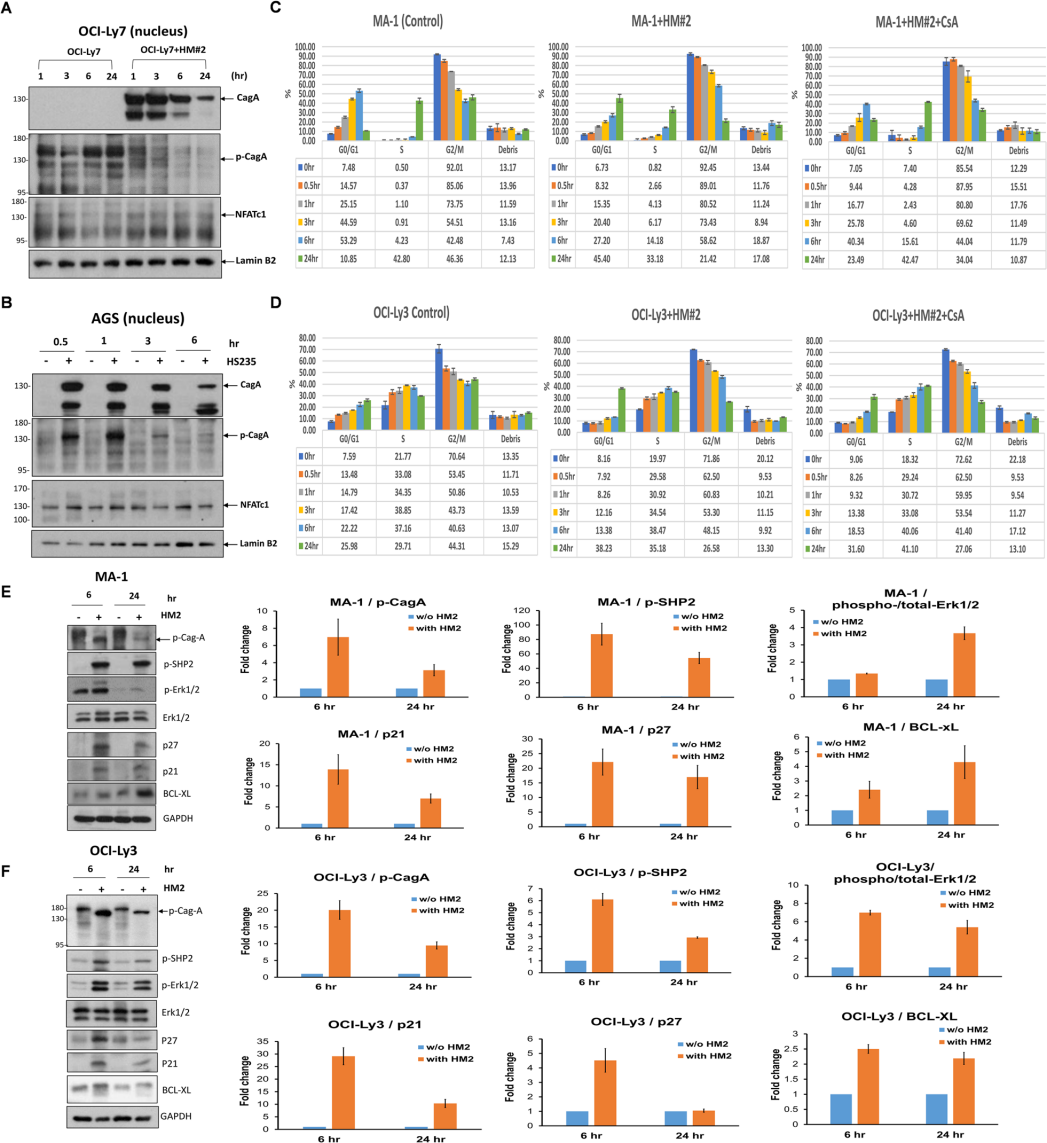
CagA, tyrosine-phosphorylated CagA, and NFATc1 can be translocated to the nucleus in HP-co-cultured GCB-DLBCL and gastric epithelial cells, CagA-related signaling molecules in HP-co-cultured DLBCL cell lines, and HP-co-cultured MA-1 cells and OCI-Ly3 cells exhibit G1 cell-cycle retardation. **A** In HP (HM#2)-co-cultured OCI-Ly7 cells, nuclear CagA expression was detected 1 and 3 h after HP stimulation; however, the effect decreased gradually at 6 h and decreased predominantly at 24 h. The expression of p-CagA in the nucleus was detected at 1 and 3 h; however, its signal intensity decreased at 6 h and eventually disappeared at 24 h. Simultaneously, the nuclear expression of NFATc1 was detected at 1, 3, 6, and 24 h after HP stimulation. **B** In HP (HS235)-co-cultured AGS cells, CagA and p-CagA translocated to the nucleus at 0.5 and 1 h. However, the signal intensity of both CagA and p-CagA decreased at 3 and 6 h. Simultaneously, nuclear expression of NFATc1 was detected at 0.5 and 1 h after HP stimulation; however, its signal intensity decreased at 3 and 6 h. **C** Compared with non-HP-co-cultured MA-1 cells, G1 arrest was predominantly detected at 24 h in HP (HM#2)-co-cultured MA-1 cells. However, G1 phase arrest was reversed after administration of CsA in HP (HM#2)-co-cultured MA-1 cells. The results were expressed in triplicate for each treatment group and measured by flow cytometry analysis (error bar means standard error). **D** Compared with that noted in non-HP-co-cultured OCI-Ly3 cells, G1 arrest was predominantly detected at 24 h in HP (HM#2)-co-cultured OCI-Ly3 cells. After the administration of CsA to HP (HM#2)-co-cultured OCI-Ly3 cells, the G1 arrest at 24 h was reversed. **E** Immunoblotting showed that HP induced the expression of CagA-related signaling molecules, including p-CagA, p-SHP2, p-ERK1/2, p21, p27, and Bcl-xL in HP (HM#2)-co-cultured MA-1 cells at 6 and 12 h. Quantification of western blotting in (**E**) showed that the expression levels of p-CagA, p-SHP2, p21, and p27 were higher at 6 than at 24 h, whereas levels of p-ERK1/2, and Bcl-xL were higher at 24 than at 6 h. **F** In HP (HM#2)-co-cultured OCI-Ly3 cells, HP provoked the expression of p-CagA, p-SHP2, p-ERK1/2, p21, p27, and Bcl-xL at 6 and at 12 h. Quantification of western blotting in (F) showed that the expression levels of p-CagA, p-SHP2, p-ERK1/2, p21, p27, and Bcl-xL were higher at 6 than at 24 h

In OCI-Ly7 (GCB-origin DLBCL) cells, nuclear CagA expression was observed at 1 and 3 h after HP stimulation. However, the signal intensity decreased gradually after 6 h and significantly at 24 h. Similarly, p-CagA expression in the nucleus was observed at 1 and 3 h. However, its signal intensity decreased significantly at 6 h and disappeared at 24 h. We also found that NFATc1 was simultaneously localized in the nucleus at 1 and 3 h, and up to 24 h (Fig. 2A).

Next, we used as controls to determine whether CagA and NFATc1 were stimulated after HP infection. CagA and p-CagA were present at higher levels in the nucleus at 0.5 and 1 h in AGS cells co-cultured with HP (HS235) than in the control (Fig. 2B). However, nuclear expression levels of CagA and p-CagA at 3 and 6 h were lower than those at 0.5 and 1 h (Fig. 2B). These findings indicate that in lymphoma B cells and gastric epithelial cells, nuclear CagA translocation occurred predominantly 1 h after HP infection; however, the level of CagA began to decline after 6 h. In contrast, the nuclear localization of NFATc1 was maintained in lymphoma B cells 24 h after HP infection, whereas it decreased 3 h after HP infection in AGS cells.

### HP infection induces cell cycle arrest of lymphoma cells via activation of CagA-related and NFATc1-dependent signals.

As NFATc1 has been reported to regulate the cell cycle inhibitors p21 and p27 [19, 42], we performed a cell proliferation assessment and cell cycle analysis of MA-1 or OCI-Ly3 cells co-cultured without HP infection, with HP infection, and with HP infection and CsA administration (Supplementary Fig. S2B).

As shown in Supplementary Fig. S2C, we found that in both MA-1 and OCI-Ly3 cells, proliferation was significantly inhibited in HP-co-cultured cells compared with that in non-HP-infected cells, whereas the inhibitory effects on proliferation were decreased after administration of CsA in HP-co-cultured B-lymphoma cells.

MA-1 (3 × 10^6^) and OCI-Ly3 (3 × 10^6^) cells were synchronized with nocodazole for 22 h, resulting in the majority of nocodazole-treated MA-1 and OCI-Ly3 cells being arrested at the G2 phase. The nocodazole was washed off and the cells were cultured and collected at the indicated time points for cell cycle analysis (Supplementary Fig. S3).

In MA-1 cells, an increased percentage of cells in the G1 phase, a decreased percentage of cells in the S phase, and an increased proportion of debris were observed immediately after 6 h of HP infection. The proportion of debris representing cell death in MA-1 cells gradually increased for up to 24 h. The G1 phase of non-HP-infected MA-1 cells decreased from 53.29% at 6 h to 10.85% at 24 h (Fig. 2C). However, the G1 phase of HP-infected MA-1 cells increased from 27.20% at 6 h to 45.40% at 24 h. However, the G1 phase decreased to 23.49% at 24 h after CsA administration in HP-infected MA-1 cells. The percentage of G2/M phase in the MA-1 cells significantly decreased 24 h after HP infection when compared with control MA-1 cells (21.42% vs. 46.36% at 24 h) (Fig. 2C). These findings indicated that HP infection can cause G1 phase arrest in MA-1 cells.

In OCI-Ly3 cells, we observed an increased percentage of cells in the G1 phase and a decreased percentage of cells in the G2/M phase 24 h after HP infection. Compared to the G1 phase (25.98%) at 24 h in the non-HP-infected OCI-Ly3 cells, the G1 phase was 38.23% at 24 h in the HP-infected OCI-Ly3 cells (Fig. 2D). The percentage of G2/M phase in OCI-Ly3 cells significantly decreased 24 h after HP infection (control vs. HP infection: 44.31% vs. 26.58%). However, G1 phase arrest was reversed after administration of CsA to HP-infected OCI-Ly3 cells (G1 phase, 31.60% at 24 h). (Fig. 2D). These findings indicated that HP infection can cause G1 phase arrest in OCI-Ly3 cells.

Next, we evaluated whether HP infection can alter the regulation of both the G1 and G2/M cell cycle regulators, p21 and p27 [43], and found that HP infection triggered the production of p-CagA and the expression levels of p-CagA were higher at 6 h than at 24 h in both MA-1 cells and OCI-Ly3 cells. Similarly, the expression levels of the NFATc1-related signaling molecules p21 and p27 were higher at 6 h than at 24 h in both MA-1 and OCI-Ly3 cells (Fig. 2E and F).

After HP co-culture, the expression levels of p-SHP-2 in both HP-co-cultured MA-1 and OCI-Ly3 cells were higher at 6 h than at 24 h (Fig. 2E and F), reflecting the significant effects of nuclear translocation of CagA and its tyrosine-phosphorylated form (p-CagA), observed from 1 to 6 h in both MA-1 and OCI-Ly3 cells (Fig. 1B and C). Furthermore, the SHP-2 regulated signaling molecule, p-ERK1/2, was higher at 24 h than at 6 h in MA-1 cells (Fig. 2E). However, p-ERK1/2 was higher at 6 h than at 24 h in OCI-Ly3 cells (Fig. 2F). Bcl-xL expression was higher at 24 h than at 6 h in HP-co-cultured MA-1 cells than in non-HP-infected MA-1 cells (Fig. 2E). We also found that Bcl-xL expression was higher in HP-co-cultured OCI-Ly3 cells than in non-HP-infected OCI-Ly3 cells (higher at 6 h than at 24 h) (Fig. 2F). These findings suggest that HP promotes the proliferation of lymphoma B cells by triggering CagA tyrosine-phosphorylation-dependent signals and limits the proliferation of lymphoma cells by activating NFATc1 and its regulation of p21/p27, thus causing G1 phase arrest in these lymphoma B cells.

### The association between expression patterns of CagA and NFATc1 molecules in lymphoma cells and HPE responsiveness of all patients

The clinicopathological features of the 59 patients with HPE-responsive (CR after first-line HPE) lymphomas and 32 patients with HPE-irresponsive lymphomas (no CR after first-line HPE) are summarized in Table 1. The median interval between completing HPE and achieving CR was 5.00 months (95% confidence interval [CI], 3.80–6.20 months; range, 1.00–22.00 months). Endoscopic appearance (gastritis-like or erosion of the infiltrative mucosa) (*p* = 0.084), distal location of the stomach (*p* = 0.073), and depth of gastric wall involvement (mucosa or submucosa) (*p* = 0.055), but not age, sex, and stage, showed an association but it was not statistically significant with HPE responsiveness. At a median follow-up of 60.70 months (95% CI, 53.75–60.75 months), 55 patients who achieved CR after HPE therapy were free of lymphoma, whereas four patients had experienced a histological relapse (relapse rate, 6.8%).

**Table 1:** Correlation of clinicopathological features and expression of CagA and NFATc1 with tumor response to HPE therapy in gastric MALT lymphoma

	Lymphomas response to HPE			
Clinicopathological characteristics	Total number (n = 91)	HPE-responsive (n = 59)	HPE-irresponsive (n = 32)	p*
Age (median, range, years)	56.0 (20–86)	57.0 (30–86)	53.5 (20–79)	0.326#
Sex, men/women	38/53	23/36	15/17	0.466§
Endoscopic features, n (%)				0.084‡
Gastritis-like or multiple erosion on infiltrative mucosa	35 (39%)	27 (46%)	8 (25%)	
Ulceration or ulcerated mass	40 (44%)	23 (39%)	17 (53%)	
Erosions on giant nodular folds	16 (17%)	9 (15%)	7 (22%)	
Location of lymphoma(s), n (%)				0.073§
Proximal^a^ or ≥ 2 components	29 (32%)	15 (25%)	14 (44%)	
Distal^b^	62 (68%)	44 (75%)	18 (56%)	
Stage				0.400§
IE	70 (77%)	47 (80%)	23 (72%)	
IIE1	21 (23%)	12 (20%)	9 (28%)	
Depth of gastric wall involvement, n (%)¶				0.055§
Submucosa or above	52/86 (61%)	38/56 (68%)	14/30 (47%)	
Muscularis propria or beyond	34/86 (39%)	18/56 (32%)	16/30 (53%)	
CagA expression				< 0.001§
Positive	54 (59%)	47 (80%)	7 (22%)	
Negative	37 (41%)	12 (20%)	25 (78%)	
Nuclear localization of NFATc1				< 0.001§
Presence	51 (56%)	43 (73%)	8 (25%)	
Absence	40 (44%)	16 (27%)	24 (75%)	
T(11;18)(q21;q21)/BIRC3-MALT1				< 0.001§
Positive	10 (11%)	0 (0%)	10 (31%)	
Negative	81 (89%)	59 (100%)	22 (69%)	

p*: comparison of discrete variables between HPE-responsive cases and HPE-irresponsive cases

^#^*p* values (two-sided) were calculated using the Student’s t-test

^§^*p* values (two-sided) were calculated using Chi-square test or Fisher’s exact test

^‡^*p* values (two-sided) were calculated using one-way analysis of variance

Proximal^a^: Middle body, upper body, fundus, or cardia. Distal^b^: Antrum, angle, or lower body

Gastric wall involvement was evaluated by endoscopic ultrasonography in 86 patients. *HPE* *Helicobacter pylori* eradication therapy, *MALT* mucosa-associated lymphoid tissue

**Fig. 3:**
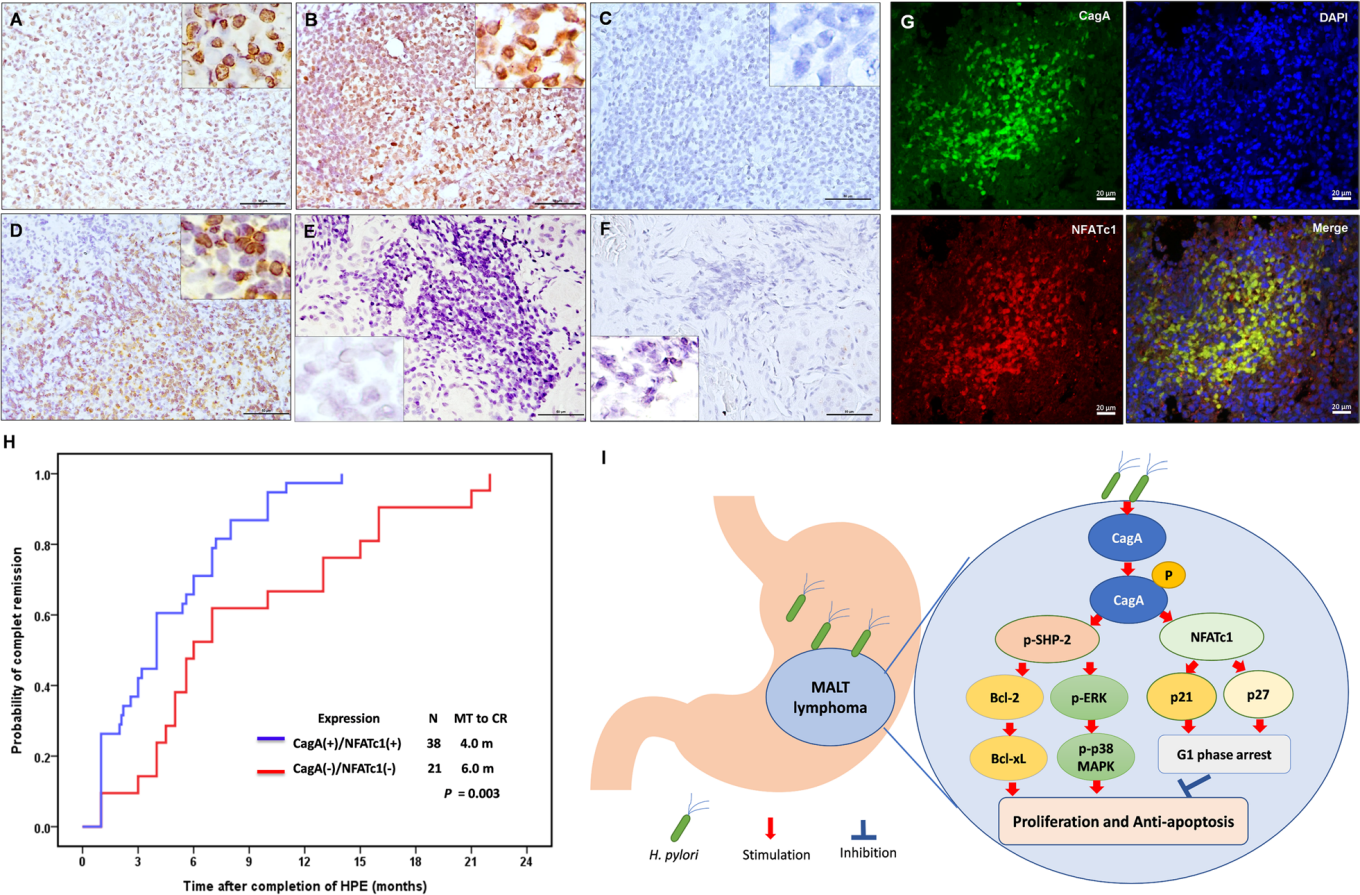
Nuclear expression pattern of NFATc1 in lymphoma cells and time to CR of patients with HPE-responsive gastric MALT lymphoma **A** Moderate nuclear localization of NFATc1 was found in the lymphoma cells of gastric mucosa in an HPE-responsive case (time to CR: 6.00 months); right upper inset, × 1000 **B** Strong nuclear localization of NFATc1 was found in the lymphoma cells of gastric mucosa in an HPE-responsive case (time to CR: 3.00 months); right upper inset, × 1000 **C** No nuclear localization of NFATc1 was found in lymphoma cells of gastric mucosa in an HPE-irresponsive case; right upper inset, × 1000 **D** A high baseline expression of nuclear localization of NFATc1 in lymphoma cells of an HPE-responsive case before HPE (case #2); right upper inset, × 1000 **E** Decreased expression of nuclear localization of NFATc1 in remitting tumor cells (case #2) 1.00 month after completion of HPE; left lower inset, × 1000 **F** No expression of nuclear localization of NFATc1 in remitting tumor cells (case #2) 4.00 months after completion of HPE (case #2, the time to CR: 7.00 month); left lower inset, × 1000 **G** Confocal laser-scanning microscopy showing that most CagA-positive cells (green fluorescence) expressed nuclear localization of NFATc1 (red fluorescence); Representative images of nucleus (stained with DAPI, blue) (right upper panel) and merged image of CagA and NFATc1 expression (right lower panel). **H** Time to CR was calculated from the completion of HPE to first evidence of CR using Kaplan–Meier analysis (CagA( +)/NFATc1( +) [both expression of CagA and nuclear NFATc1 localization in lymphoma cells] vs. CagA(−)/NFATc1(−) [either CagA or nuclear NFATc1localization, or absence expression of CagA and nuclear NFATc1 localization in lymphoma cells]; two-sided log-rank test; *p* = 0.003). **I** In the stomach, persistent HP infection can result in the translocation of HP-encoded CagA into B lymphocytes and trigger the tyrosine phosphorylation-dependent signaling pathway, including SHP-2 and its ERK, p38 MAPK, Bcl-2, and Bcl-xL. This CagA-regulated signaling pathway further promotes the proliferation and impedes the apoptosis of lymphoma B cells. Simultaneously, CagA stimulates the production of NFATc1, which further upregulates the expression of p21 and p27, restricting cell cycle progression to the G1 phase and limiting the growth of lymphoma B cells. *N* number, *MT* median time, *CR* complete remission

We observed the presence of CagA in lymphoma cells in 54 (59%) of the 91 patients, and the presence of CagA was significantly correlated with HPE responsiveness (47/59 [80%] HPE-responsive cases vs. 7/32 [22%] HPE-irresponsive cases, *p* < 0.001; Table 1). Nuclear localization of NFATc1 was detected (29 cases, score 2; 22 cases, score 3) in 51 (56%) of the 91 patients (Fig. 3). The remaining 40 patients tested negative for nuclear localization of NFATc1 (35 patients, score 0; 5 patients, score 1). Nuclear localization of NFATc1 was significantly higher in HPE-responsive cases than in HPE-irresponsive cases (73% [43/59] vs. 25% [8/32]; *p* < 0.001) (Table 1). As shown in Supplementary Fig. S4, nuclear localization of CagA was observed in lymphoma cells of the gastric mucosa, and nuclear localization of NFATc1 was observed in the same regions of lymphoma cells expressing CagA. We used CLSM to confirm that CagA and NFATc1 colocalized in the nuclei of HPE-responsive gastric MALT lymphoma cells (Fig. 3).

We also assessed serial changes in the nuclear localization of NFATc1 in lymphoma cells before and after HPE completion in four patients with HPE-responsive gastric MALT lymphoma. Nuclear localization of NFATc1 was significantly downregulated in the lymphoma cells of two patients (cases #1 and #2) who achieved partial remission and CR at 4.00 and 7.00 months, respectively, after the completion of HPE (Fig. 3). We detected no nuclear localization of NFATc1 in gastric biopsies or remitting lymphoma cells from two other patients (cases #3 and #4) who achieved CR 1.00 and 4.00 months after completing HPE.

Table 2 shows the demographic characteristics of the two groups of patients (presence or absence of nuclear localization of NFATc1) and their clinicopathological features, including age, sex, endoscopic appearance, and lesion site. The table shows non-significant differences between the two groups. Moreover, the nuclear localization of NFATc1 was closely associated with the presence of CagA (Spearman correlation coefficient = 0.484, *p* < 0.001) (Table 2). Co-expression of CagA and nuclear NFATc1 was found in only one (10%) of the patients harboring t(11;18)(q21;q21), whereas t(11;18)(q21;q21) was exclusively detected in patients with HPE irresponsiveness (Table 1).

**Table 2:** Clinicopathological features and NFATc1 expression in patients with stage IE/IIE1 gastric MALT lymphoma who received first-line HPE therapy

	Nuclear localization of NFATc1				
Clinicopathological characteristics	Total number (n = 91)	Presence (n = 51)	Absence (n = 40)	p*	
Age (median, range, years)	56.0 (20–86)	57.0 (30–83)	55.0 (20–86)	0.608#	
Sex, men/women	38/53	21/30	17/23	0.899§	
Endoscopic features, n (%)				0.289‡	
Gastritis-like or multiple erosion on infiltrative mucosa	35 (38%)	19 (37%)	16 (40%)		
Ulceration or ulcerated mass	40 (44%)	20 (39%)	20 (50%)		
Erosions on giant nodular folds	16 (18%)	12 (24%)	4 (10%)		
Location of lymphoma(s), n (%)				0.570§	
Proximal^a^ or ≥ 2 components	29 (32%)	15 (29%)	14 (35%)		
Distal^b^	62 (68%)	36 (71%)	26 (65%)		
Stage				0.537§	
IE	70 (77%)	38 (75%)	32 (80%)		
IIE1	21 (23%)	13 (25%)	8 (20%)		
Depth of gastric wall involvement, n (%)¶				0.380§	
Submucosa or above	52/86 (60%)	31/48 (65%)	21/38 (55%)		
Muscularis propria or beyond	34/86 (40%)	17/48 (35%)	17/38 (45%)		
CagA expression				< 0.001§	
Positive	54 (59%)	41 (80%)	13 (33%)		
Negative	37 (41%)	10 (20%)	27 (67%)		
T(11;18)(q21;q21)/BIRC3-MALT1				0.002	
Positive	10 (11%)	1 (2%)	9 (23%)		
Negative	81 (89%)	50 (98%)	31 (77%)		

p*: comparison of discrete variables between NFATc1-positive cases and NFATc1-negative cases

^#^*p* values (two-sided) were calculated using the Student’s t-test

^§^*p* values (two-sided) were calculated using Chi-square test or Fisher’s exact test

^‡^*p* values (two-sided) were calculated using one-way analysis of variance

Proximal^a^: Middle body, upper body, fundus, or cardia. Distal^b^: Antrum, angle, or lower body

Gastric wall involvement was evaluated by endoscopic ultrasonography in 86 patients

*HPE* *Helicobacter pylori* eradication therapy, *MALT* mucosa-associated lymphoid tissue

Multivariate analysis identified the presence of CagA (*p* < 0.001) as an independent marker for predicting HPE responsiveness in gastric MALT lymphoma, and nuclear localization of NFATc1 (*p* = 0.053) showed a non-significant association with HPE responsiveness in gastric MALT lymphoma. However, the endoscopic appearance of gastritis-like or multiple erosions (*p* = 0.141), lymphomas located in the distal part of the stomach (*p* = 0.301), and lymphomas with mucosal or submucosal involvement (*p* = 0.177) were not associated with HPE responsiveness of gastric MALT lymphoma (Table 3).

**Table 3:** Association of clinicopathologic factors and expression of CagA and nuclear localization of NFATc1 with HPE responsiveness of gastric MALT lymphoma using multivariate analyses

Variables	Odds Ratio	95% confidence interval	*p* value
All patients with gastric MALT lymphoma (n = 91)			
Lymphomas Location: Distal vs. proximal or ≧ 2 components	1.887	0.567–6.282	0.301
Endoscopic features: Gastritis-like or multiple erosion vs ulceration or mass, or giant modular folds	2.706	0.720–10.177	0.141
Depth of gastric involvement: Submucosa or above vs. muscularis or beyond	2.283	0.689–7.558	0.177
CagA expression vs. no CagA expression	11.905	3.314–42.767	< 0.001
Presence of nuclear NFATc1 localization vs. absence of nuclear NFATc1 localization	3.524	0.986–12.595	0.053
Patients with gastric MALT lymphoma who had no t(11;18)(p21;q21)s (n = 81)			
Lymphomas Location: Distal vs. proximal or ≧ 2 components	1.841	0.461–7.353	0.388
Endoscopic features: Gastritis-like or multiple erosion vs ulceration or mass, or giant modular folds	5.703	1.069–30.430	0.042
Depth of gastric involvement: Submucosa or above vs. muscularis or beyond	3.374	0.939–14.840	0.061
CagA expression vs. no CagA expression	12.616	2.904–54.800	0.001
Presence of nuclear NFATc1 localization vs. absence of nuclear NFATc1 localization	3.564	0.833–15.243	0.086

When CagA and NFATc1 were used as single markers to predict HPE responsiveness in gastric MALT lymphoma, the positive predictive values (PPV) for CagA and NFATc1 were 87.0% and 84.3%, respectively, and the specificity values for CagA and NFATc1 were 78.1% and 75.0%, respectively (Table 4). Among the 59 HPE-responsive patients, 38 expressed CagA and NFATc1. The combination of CagA and nuclear NFATc1 localization showed increased PPV (90.5%) and specificity (87.5%) for HPE responsiveness compared with CagA expression or nuclear localization of NFATc1 alone (Table 4). Among 59 HPE-responsive cases, patients with both expression of CagA and NFATc1 responded to HPE more rapidly than those without the expression of both CagA and nuclear NFATc1 localization (median interval to CR after completing HPE, 4.00 months (95% CI, 3.21–4.79 months) vs. 6.00 months (95% CI, 4.21–7.80 months), *p* = 0.003, log-rank test) (Fig. 3H).

**Table 4:** Positive predictive values of CagA and NFATc1 for HPE responsiveness in stage IE/IIE1 gastric MALT lymphoma

Expression of CagA and NFATc1 molecules in tumor tissue	Number of patients with gastric MALT lymphoma who received first-line HPE	
	HPE-responsive (n = 59)	HPE-irresponsive (n = 32)
CagA positive	47	7
CagA negative	12	25
PPV^a^ for CagA expression = 47 of 54 (87.0%)		
Specificity^b^ for CagA expression = 25 of 32 (78.1%)		
NFATc1 positive	43	8
NFATc1 negative	16	24
PPV^a^ for NFATc1 expression = 43 of 51 (84.3%)		
Specificity^b^ for NFATc1 expression = 24 of 32 (75.0%)		
CagA and NFATc1 are all positive		
Yes	38	4
No	21	28
PPV¶ for combined CagA and NFATc1 = 38 of 42 (90.5%)		
Specificity^c^ for combined CagA and NFATc1 = 28 of 32 (87.5%)		

^a^Positive predictive value (PPV) = Number of HPE-responsive cases who had CagA expression or nuclear NFATc1 localization/Total positive cases for respective CagA expression or nuclear NFATc1 localization

^b^Specificity = Number of HPE-irresponsive cases who had no CagA expression or nuclear NFATc1 localization/Total HPE-irresponsive cases

PPV = Number of HPE-responsive cases who had all CagA and nuclear NFATc1 localization/Total cases expressing all CagA and nuclear NFATc1 localization

^c^Specificity = Number of HPE-irresponsive cases who did not simultaneously express CagA and nuclear NFATc1 localization in tumors/Total HPE-irresponsive cases

*MALT* Mucosa-associated lymphoid tissue, *HPE* *Helicobacter pylori* eradication

### Correlation of presence of CagA and nuclear localization of NFATc1 in lymphoma cells with HPE responsiveness of gastric MALT lymphoma patients without t(11;18)(p21;q21)

Of the 91 patients with gastric MALT lymphoma, ten had t(11;18)(p21, q21)- and HPE-irresponsive lymphomas. In the remaining 81 patients, the lymphomas were negative for t(11;18)(p21;q21); 59 patients had HPE-responsive lymphomas, and 22 had HPE-irresponsive lymphomas. As shown in Supplementary Table S1, the endoscopic appearances of gastritis-like or multiple erosions (*p* = 0.029), lymphomas with involvement of the mucosa or submucosa (*p* = 0.010), the presence of CagA (*p* < 0.001), and nuclear localization of NFATc1 (*p* < 0.001) were significantly correlated with HPE responsiveness of these tumors, whereas lymphomas located at the gastric distal part (*p* = 0.083) were associated, though not significantly, with HPE responsiveness of these patients without t(11;18)(p21;q21).

Multivariate analysis also showed that the endoscopic appearance of gastritis-like or multiple erosions (*p* = 0.042) and the presence of CagA (*p* = 0.001) were independent predictors of HPE responsiveness for these lymphomas. Lymphomas with involvement of the mucosa or submucosa (*p* = 0.061) and nuclear localization of NFATc1 (*p* = 0.086) were associated, though not significantly, with HPE responsiveness for gastric MALT lymphoma without t(11;18)(p21;q21). However, lymphomas located in the distal gastric region (*p* = 0.388) did not correlate with HPE responsiveness (Table 3).

In patients without t(11;18)(q21;q21), nuclear localization of NFATc1 was significantly associated with the presence of CagA (Spearman correlation coefficient = 0.389; *p* < 0.001) but was not associated with other clinicopathological features (Supplementary Table S2). Compared with CagA expression or nuclear localization of NFATc1 alone, the combination of CagA expression and nuclear NFATc1 localization showed increased PPV (92.7%) and specificity (86.4%) for HPE responsiveness in these lymphomas (Supplementary Table S3).

## Discussion

In this study, CagA translocated from HP promoted cell proliferation and inhibited cell cycle progression through tyrosine phosphorylation-dependent and NFATc1-regulated signaling, respectively, and contributed to CR of lymphoma if HP was successfully eradicated. This biological significance was further confirmed in tumor samples from patients with gastric MALT lymphoma, in which the nuclear localization of NFATc1 significantly correlated with CagA expression in lymphoma cells and the CR of these tumors. This study identified the cooperative contribution of CagA and NFATc1 in the HPE-responsive pathogenesis of gastric MALT lymphoma.

Previous studies have shown that the CagA protein may be translocated to B lymphocytes and further trigger a waterfall in survival signaling for the promotion of B lymphocytes when HP contact gastric epithelial surfaces [44, 45]. By studying gastric epithelial cells, it was reported that the biological half-life of CagA is approximately 200 min, during which the stability of CagA is dependent on its interaction with partitioning-defective 1 [46]. In three types of lymphoma B cells (MA-1, OCI-Ly3, and OCI-Ly7), translocated tyrosine-phosphorylated CagA was initially detected 1 h after HP infection and decreased at 6 h after HP infection, whereas in gastric epithelial cells, tyrosine-phosphorylated CagA decreased at 3 h after HP infection. These findings indicated that the half-life of CagA is approximately 3 h. Based on these findings, we postulated that the proliferation of lymphoma B cells is primarily dependent on CagA and its tyrosine phosphorylation-dependent signaling molecules (p-SHP-2, p-ERK, and Bcl-xL). The short biological half-life of CagA in B lymphocytes may explain why HPE leads to a CR in lymphoma cells, particularly in CagA-positive gastric MALT lymphoma cases [17, 40, 47, 48]. As CagA cannot continuously regulate the SHP-2-dependent signaling pathway to promote tumor cell growth, regression of lymphoma cells is observed once HP is eradicated [16, 49, 50].

Expression of CagA in gastric epithelial cells leads to NFAT translocation from the cytoplasm to the nucleus through activation of the calcineurin signaling pathway, where nuclear translocation of NFAT further activates *p21*, resulting in G1 arrest in these epithelial cells [19]. Our current findings indicate that the nuclear translocation of NFATc1 following CagA tyrosine phosphorylation after HP infection activates NFATc1-dependent genes such as *p21* and *p27* in both B-cell lymphoma cell lines (MA-1 and OCI-Ly3) [19, 43]. These two elevated G1-checkpoint CDK inhibitors (p21 and p27) arrested both MA-1 and OCI-Ly3 cells in the G1 phase [51–54]. These important biological functions of NFATc1 in limiting tumor progression in lymphoma B cells are in line with our current immunohistochemical findings showing a significant association between the nuclear localization of NFATc1, CagA, and HPE responsiveness in gastric MALT lymphoma. In addition, among these HPE-responsive gastric MALT lymphomas, nuclear localization of NFATc1 was downregulated in post-HPE gastric biopsy samples. Previous studies have demonstrated that NFATc1 is involved in intracellular signaling in B-lymphoid cells [20–22, 55, 56]. In a type I Burkitt's lymphoma cell line study, NFAT/calcineurin signaling promoted B-cell antigen receptor-mediated apoptosis [57], whereas in gastric lymphoma samples, p27 (Kip1) was exclusively expressed in MALT lymphoma cells, but not in DLBCL lymphoma cells, with or without histological evidence of MALT lymphoma [58].

CagA and NFATc1 colocalized in the lymphoma cells of patients with HPE-responsive gastric MALT lymphoma, and the combined presence of CagA and nuclear localization of NFATc1 increased the PPV (90.5%) and specificity (87.5%) for HPE responsiveness compared with CagA expression alone. Furthermore, patients with both CagA expression and nuclear localization of NFATc1 in tumor cells responded to HPE more rapidly than those with either CagA or NFATc1 expression alone, or with no expression of CagA or NFATc1. Most CagA molecules in the HP strains from East Asian gastric MALT lymphomas show greater tyrosine phosphorylation activity and SHP-2 binding affinity for the EPIYA-D motif [59–61]. These findings suggest that the CagA-tyrosine phosphorylation-dependent signaling pathway contributes to the growth of HPE-responsive gastric MALT lymphoma and that CagA-stimulated NFATc1 signaling further restricts the progression of these tumors by activating the CDK inhibitors p21 and p27 (Fig. 3I) [48, 62].

In gastric epithelial cells, Yokoyama et al. showed that the HP-encoded CagA protein triggers the activation of NFATc3 (from the cytoplasm to the nucleus); however, another HP oncoprotein, VacA, impedes the nuclear translocation of NFATc3 and the subsequent production of p21 [19]. Previous studies have shown that VacA reduces NFAT activity in T lymphocytes, hampering the growth and immune response of T lymphocytes and interleukin (IL)-2 cytokine production [63, 64]. Although we did not determine whether VacA could inhibit the activation of NFATc1 by CagA in lymphoma B cells, our findings showed that CagA, but not VacA, was stimulated by HP in HP-co-cultured B lymphoma cells. We further found that nuclear NFATc1 expression was significantly higher in HPE-responsive than in HPE-irresponsive MALT lymphoma of the stomach and was significantly associated with the expression of CagA. These findings suggest that HP stimulates the production of CagA and its regulated signaling pathway, promotes the proliferation and progression of B-cell lymphoma B cells, and constrains the progression of these lymphoma cells through NFATc1 signaling. This mechanism may explain why most HP-positive gastric MALT lymphomas are localized to the stomach and rarely disseminate distantly.

In addition to seropositivity for CagA, patients with gastric MALT lymphoma have been reported to be positive for HP CagY, a virB10-homologous protein, which may alter the function of the type IV secretion system and interfere with the injection of CagA into gastric epithelial or B cells [65, 66]. Recently, Della Bella et al. revealed that 22 (14%) CD4 + T-cell clones from 158 patients with MALT lymphoma and three (2%) CD4 + T-cell clones from 179 patients with chronic gastritis reacted to HP CagY [67]. However, CD8 + T-cell clones obtained from patients with MALT lymphoma or chronic gastritis did not react with HP CagY [67]. In their study, CagY-specific CD4 + clones from patients with gastric MALT lymphoma promoted the proliferation of B cells by producing IFN-γ and IL-17 (produced by T helper 17 [Th17] cells) [67]. We previously reported that gastric tissue expression of IL-22, a Th17-related cytokine, was significantly associated with HPE responsiveness in patients with gastric MALT lymphoma [68]. However, whether CagY modulates the translocation of CagA to B cells in gastric MALT lymphoma remains unclear.

## Conclusions

Our current study showed that the nuclear localization of NFATc1 in lymphoma cells significantly correlated with the presence of CagA in lymphoma cells and HPE responsiveness in patients with gastric MALT lymphoma. The biological significance of nuclear localization of NFATc1 is accompanied by the activation of CDK inhibitors (p21 and p27), which contribute to G1 arrest in lymphoma B cells. Further investigations into the molecular mechanisms underlying the cooperative participation of CagA and NFATc1 in the pathogenesis of HPE-responsive gastric lymphoma are warranted.

## Supplementary Information

Supplementary material 1. Fig. S1. CagA and NFATc1 translocate to the nucleus in HP-co-cultured lymphoma B-cells (A) The time point of assay to detect the localization of NFATc1 after co-culture of B-lymphoma cells (MA-1, MA-1#46, OCI-Ly3, and OCI-Ly7) with HP strains (B) In HP (HM#12)-co-cultured MA-1 cell, nuclear expression of CagA was predominantly found at 0.5, 1, 3, and 6 h after HP infection. In addition, nuclear expression of NFATc1 was found at 0.5 h, increased gradually, and became predominant at 1, 3, and 6 h after HP. However, CsA treatment downregulated nuclear NFATc1 expression at 0.5, 1, 3, and 6 h after HP infection but did not affect nuclear CagA expression. The expression of nuclear VacA was not obvious in HP (HM#12)-co-cultured MA-1 cell compared with non-HP-co-cultured MA-1 cells. In addition, CsA administration did not alter nuclear VacA expression. HP, *Helicobacter pylori*; MOI, multiplicity of infection; PBS, phosphate-buffered saline; IFC, immunofluorescence; CagA, cytotoxin-associated gene A; NFAT, nuclear factor of activated T cells; CsA, cyclosporine A. VacA, vacuolating toxin A.

Supplementary material 2. Fig. S2. (A) We used 1, 0.5, 0.25, 0.125, and 0 mg/L clarithromycin (CAM) to treat HP on culture plates. The inhibitory effect of CAM on HP growth was dose-dependent. The data of inhibitory effects of CAM in the growth of HP is shown in the lower panel (the results are expressed for triplicates in each treatment group; error bar means standard errors). (B) The time point of assay and the schedule to assess the proliferation and cell cycles of MA-1 and OCI-Ly3 cells which were co-cultured without HP infection, with HP infection, and with HP infection and cyclosporine A (CsA) (C) The results of proliferation of MA-1 and OCI-Ly3 cells co-cultured without HP, with HP infection, and with HP infection and cyclosporine A (CsA) (the results are expressed for triplicates in each treatment group, error bar means standard errors).

Supplementary material 3. Fig. S3. (A) The MA-1 cells and OCI-Ly3 cells were synchronized with nocodazole, resulting in most cells being arrested at the G2 phase and serving as controls. (B) Flow cytometric analysis of cell cycle distribution in MA-1 cells (upper panel) and OCI-Ly3 cells (lower panel) co-cultured with or without the HP strain, and the HP strain and cyclosporine A (CsA).

Supplementary material 4. Fig. S4. Examples of expression patterns of CagA and NFATc1 in tumor cells of HPE-responsive gastric MALT lymphoma (A) CagA expression was found in the tumor cells of gastric mucosa or submucosa in three sample cases of HPE-responsive gastric MALT lymphoma (case 1#, case 3#, and case 4#) (all images ×400) (B) Nuclear NFATc1 expression was found in the same cases of tumor cells of gastric mucosa or submucosa. (case 1#, case 3#, and case 4#) (all images ×400). CagA, cytotoxin-associated gene A; NFAT, nuclear factor of activated T cells; HP, *Helicobacter pylori*; MALT, mucosa-associated lymphoid tissue.

Supplementary material 5.

Supplementary material 6. Table S1. Correlation of clinicopathological features and expression of CagA and NFATc1 with tumor response to HPE therapy in gastric MALT lymphoma without t(11;18)(p21;q21).

Supplementary material 7. Table S2. Clinicopathological features and NFATc1 expression in patients with stage IE/IIE1 gastric MALT lymphoma without t(11;18)(p21;q21) who received first-line HPE therapy.

Supplementary material 8. Table S3. Positive predictive values of CagA and NFATc1 for HPE responsiveness in stage IE/IIE1 gastric MALT lymphoma without t(11;18)(p21;q21).

## Data Availability

No datasets were generated or analysed during the current study.

## Abbreviations

ABC: Activated B-cell-like
Bcl-2: B-cell lymphoma 2
Bcl-xL: B-cell lymphoma-extra-large
CagA: Cytotoxin-associated gene A
CDK: Cyclin-dependent kinase
CI: Confidence interval
CLSM: Confocal laser-scanning microscopy
CR: Complete remission
CsA: Cyclosporine A
DAPI: 4’,6-Diamidino-2-phenylindole
DLBCL: Diffuse large B-cell lymphoma
EPIYA: Glutamic acid-proline-isoleucine-tyrosine-alanine
ERK: Extracellular signal-regulated kinase
FBS: Fetal bovine serum
GCB: Germinal center B-like
HP: Helicobacter pylori
MALT: Mucosa-associated lymphoid tissue
MAPK: Mitogen-activated protein kinase
MOI: Multiplicity of infection
SHP-2: Src homology-2 domain-containing phosphatase
NFAT: Nuclear factor of activated T cells
P: Phospho
PAGE: Glycine polyacrylamide gel electrophoresis
PBS: Phosphate-buffered saline
PPV: Positive predictive value
PVDF: Polyvinylidene difluoride
RPMI: Roswell Park Memorial Institute
SDS: Sodium dodecyl sulfate
STR: Short tandem repeat
Th: T helper
VacA: Vacuolating cytotoxin A
