# Supplementary material for: Cooperative participation of CagA and NFATc1 in the pathogenesis of antibiotics-responsive gastric MALT lymphoma
Source: Cancer Cell Int. 2024 Nov 18;24:383. doi: 10.1186/s12935-024-03552-6 (PMC11575159; doi:10.1186/s12935-024-03552-6)
Supplement: Supplementary file 1 — Supplementary material 1. Fig. S1. CagA and NFATc1 translocate to the nucleus in HP-co-cultured lymphoma B-cells (A) The time point of assay to detect the localization of NFATc1 after co-culture of B-lymphoma cells (MA-1, MA-1#46, OCI-Ly3, and OCI-Ly7) with HP strains (B) In HP (HM#12)-co-cultured MA-1 cell, nuclear expression of CagA was predominantly found at 0.5, 1, 3, and 6 h after HP infection. In addition, nuclear expression of NFATc1 was found at 0.5 h, increased gradually, and became predominant at 1, 3, and 6 h after HP. However, CsA treatment downregulated nuclear NFATc1 expression at 0.5, 1, 3, and 6 h after HP infection but did not affect nuclear CagA expression. The expression of nuclear VacA was not obvious in HP (HM#12)-co-cultured MA-1 cell compared with non-HP-co-cultured MA-1 cells. In addition, CsA administration did not alter nuclear VacA expression. HP, Helicobacter pylori; MOI, multiplicity of infection; PBS, phosphate-buffered saline; IFC, immunofluorescence; CagA, cytotoxin-associated gene A; NFAT, nuclear factor of activated T cells; CsA, cyclosporine A. VacA, vacuolating toxin A. [file 12935_2024_3552_MOESM1_ESM.pdf]

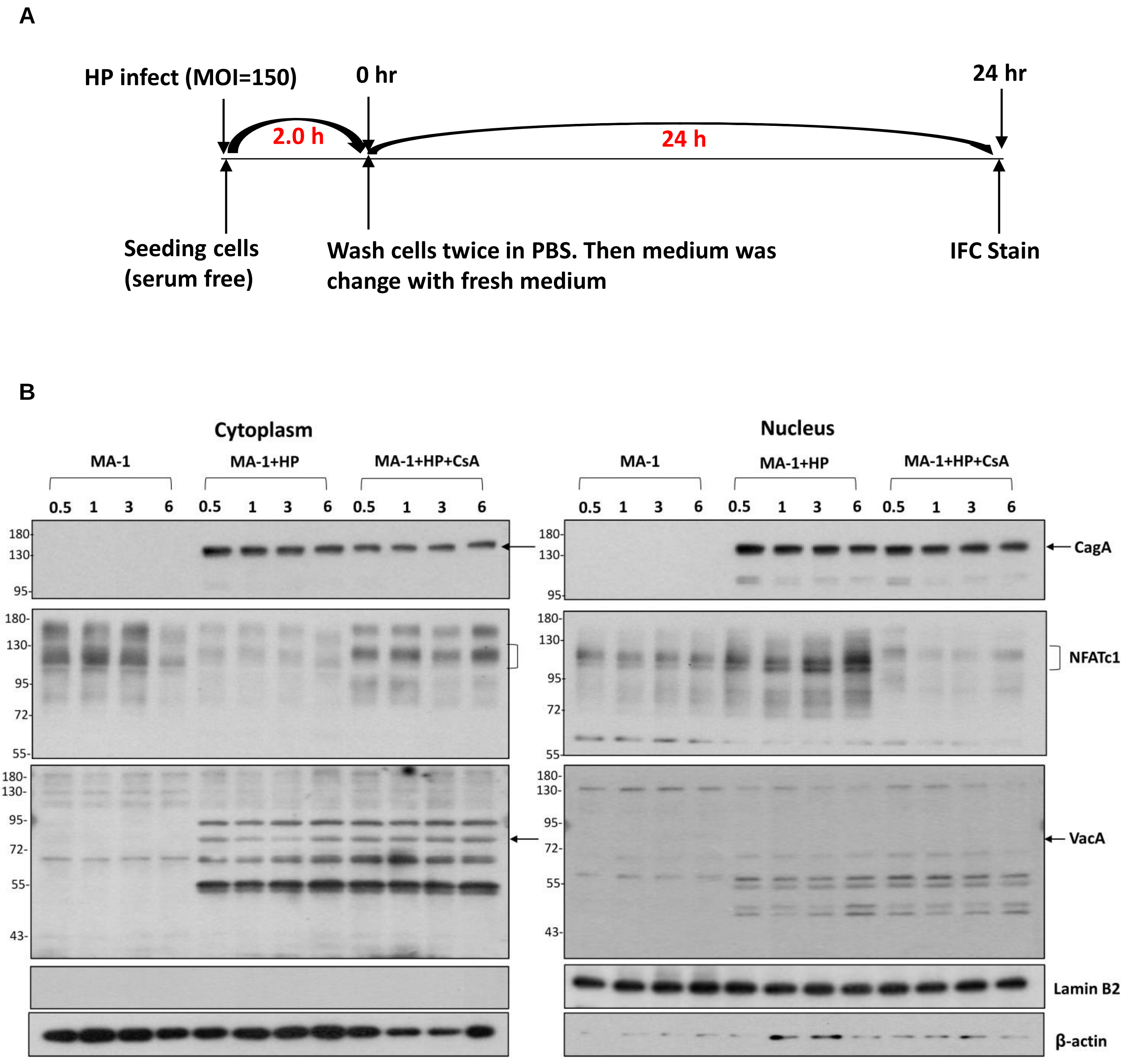

Supplementary Fig. S1. CagA and NFATc1 translocate to the nucleus in HP-co-cultured lymphoma B-cells (A) The time point of assay to detect the localization of NFATc1 after co-culture of B-lymphoma cells (MA-1, MA-1#46, OCI-Ly3, and OCI-Ly7) with HP strains (B) In HP (HM#12)-co-cultured MA-1 cell, nuclear expression of CagA was predominantly found at 0.5, 1, 3, and 6 h after HP infection. In addition, nuclear expression of NFATc1 was found at 0.5 h, increased gradually, and became predominant at 1, 3, and 6 h after HP. However, CsA treatment downregulated nuclear NFATc1 expression at 0.5, 1, 3, and 6 h after HP infection but did not affect nuclear CagA expression. The expression of nuclear VacA was not obvious in HP (HM#12)-co-cultured MA-1 cell compared with non-HP-co-cultured MA-1 cells. In addition, CsA administration did not alter nuclear VacA expression. HP, *Helicobacter pylori*; MOI, multiplicity of infection; PBS, phosphate-buffered saline; IFC, immunofluorescence; CagA, cytotoxin-associated gene A; NFAT, nuclear factor of activated T cells; CsA, cyclosporine A. VacA, vacuolating toxin A.
