# Supplementary material for: Cooperative participation of CagA and NFATc1 in the pathogenesis of antibiotics-responsive gastric MALT lymphoma
Source: Cancer Cell Int. 2024 Nov 18;24:383. doi: 10.1186/s12935-024-03552-6 (PMC11575159; doi:10.1186/s12935-024-03552-6)
Supplement: Supplementary file 2 — Supplementary material 2. Fig. S2. (A) We used 1, 0.5, 0.25, 0.125, and 0 mg/L clarithromycin (CAM) to treat HP on culture plates. The inhibitory effect of CAM on HP growth was dose-dependent. The data of inhibitory effects of CAM in the growth of HP is shown in the lower panel (the results are expressed for triplicates in each treatment group; error bar means standard errors). (B) The time point of assay and the schedule to assess the proliferation and cell cycles of MA-1 and OCI-Ly3 cells which were co-cultured without HP infection, with HP infection, and with HP infection and cyclosporine A (CsA) (C) The results of proliferation of MA-1 and OCI-Ly3 cells co-cultured without HP, with HP infection, and with HP infection and cyclosporine A (CsA) (the results are expressed for triplicates in each treatment group, error bar means standard errors). [file 12935_2024_3552_MOESM2_ESM.pdf]

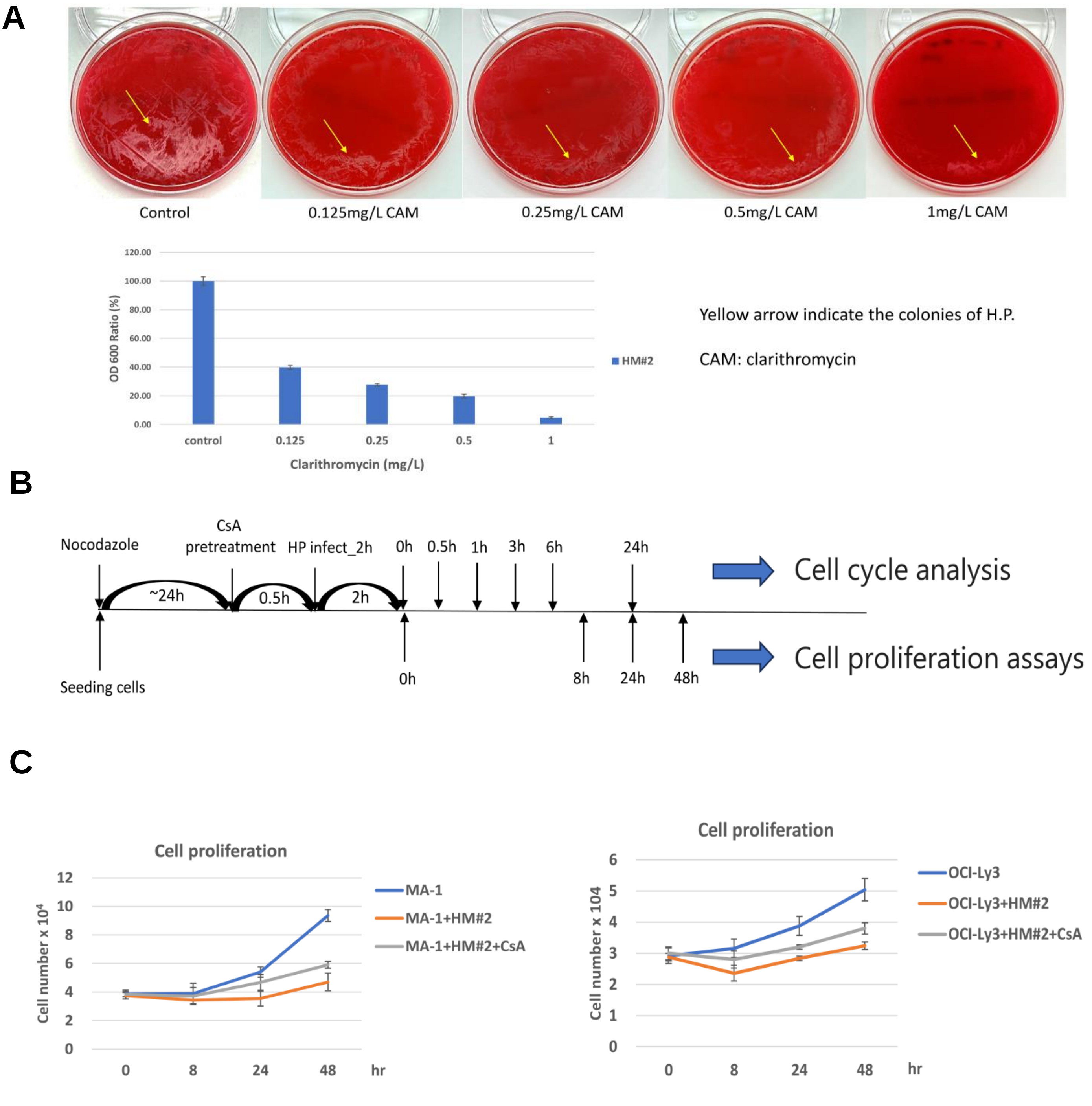

Supplementary Fig. S2. (A) We used 1, 0.5, 0.25, 0.125, and 0 mg/L clarithromycin (CAM) to treat HP on culture plates. The inhibitory effect of CAM on HP growth was dose-dependent. The data of inhibitory effects of CAM in the growth of HP is shown in the lower panel (the results are expressed for triplicates in each treatment group; error bar means standard errors). (B) The time point of assay and the schedule to assess the proliferation and cell cycles of MA-1 and OCI-Ly3 cells which were co-cultured without HP infection, with HP infection, and with HP infection and cyclosporine A (CsA) (C) The results of proliferation of MA-1 and OCI-Ly3 cells co-cultured without HP, with HP infection, and with HP infection and cyclosporine A (CsA) (the results are expressed for triplicates in each treatment group, error bar means standard errors).
