# Supplementary material for: Cooperative participation of CagA and NFATc1 in the pathogenesis of antibiotics-responsive gastric MALT lymphoma
Source: Cancer Cell Int. 2024 Nov 18;24:383. doi: 10.1186/s12935-024-03552-6 (PMC11575159; doi:10.1186/s12935-024-03552-6)
Supplement: Supplementary file 3 — Supplementary material 3. Fig. S3. (A) The MA-1 cells and OCI-Ly3 cells were synchronized with nocodazole, resulting in most cells being arrested at the G2 phase and serving as controls. (B) Flow cytometric analysis of cell cycle distribution in MA-1 cells (upper panel) and OCI-Ly3 cells (lower panel) co-cultured with or without the HP strain, and the HP strain and cyclosporine A (CsA). [file 12935_2024_3552_MOESM3_ESM.pdf]

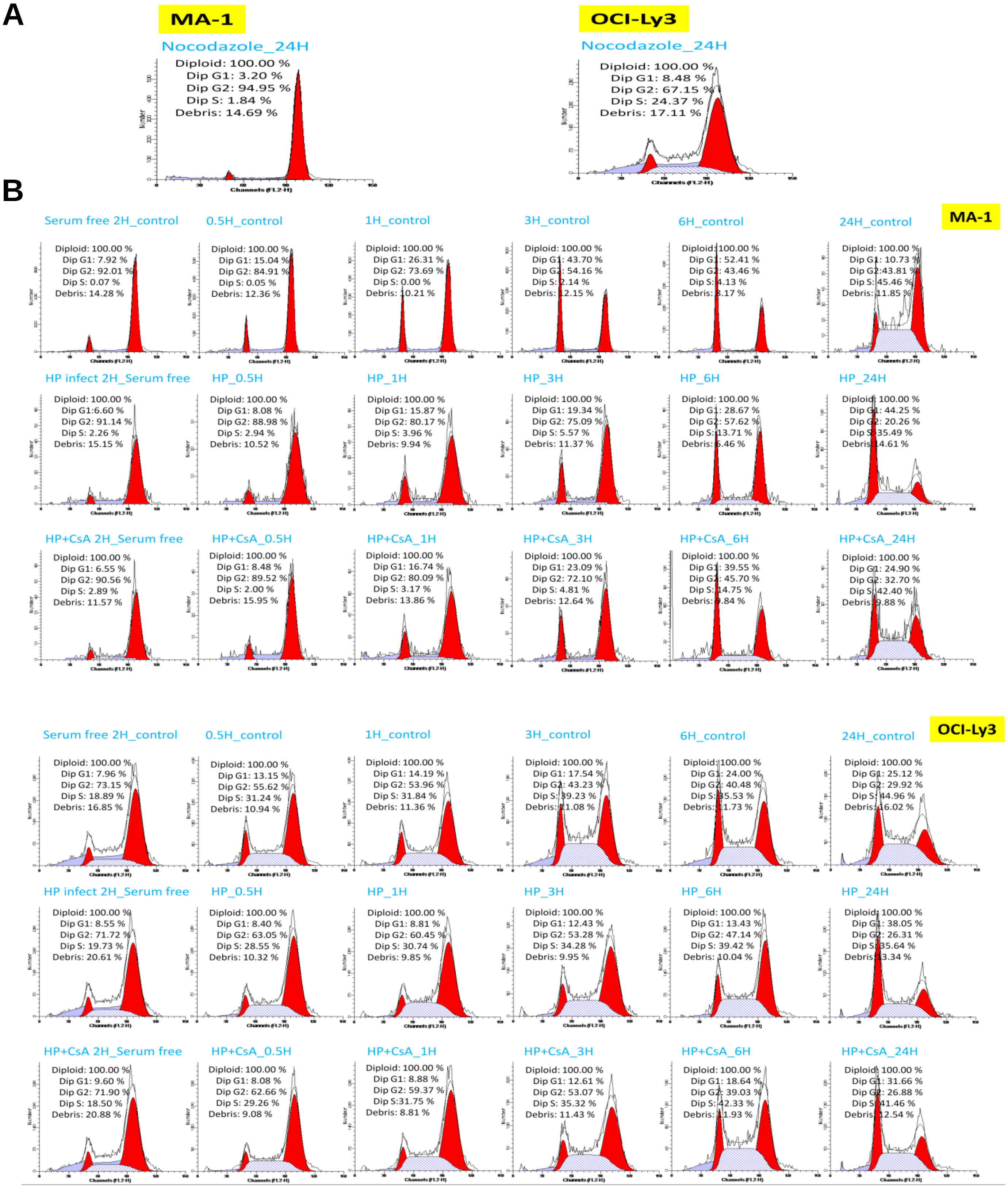

Supplementary Fig. S3. (A) The MA-1 cells and OCI-Ly3 cells were synchronized with nocodazole, resulting in most cells being arrested at the G2 phase and serving as controls. (B) Flow cytometric analysis of cell cycle distribution in MA-1 cells (upper panel) and OCI-Ly3 cells (lower panel) co-cultured with or without the HP strain, and the HP strain and cyclosporine A (CsA).
