# Supplementary material for: Cooperative participation of CagA and NFATc1 in the pathogenesis of antibiotics-responsive gastric MALT lymphoma
Source: Cancer Cell Int. 2024 Nov 18;24:383. doi: 10.1186/s12935-024-03552-6 (PMC11575159; doi:10.1186/s12935-024-03552-6)
Supplement: Supplementary file 4 — Supplementary material 4. Fig. S4. Examples of expression patterns of CagA and NFATc1 in tumor cells of HPE-responsive gastric MALT lymphoma (A) CagA expression was found in the tumor cells of gastric mucosa or submucosa in three sample cases of HPE-responsive gastric MALT lymphoma (case 1#, case 3#, and case 4#) (all images ×400) (B) Nuclear NFATc1 expression was found in the same cases of tumor cells of gastric mucosa or submucosa. (case 1#, case 3#, and case 4#) (all images ×400). CagA, cytotoxin-associated gene A; NFAT, nuclear factor of activated T cells; HP, Helicobacter pylori; MALT, mucosa-associated lymphoid tissue. [file 12935_2024_3552_MOESM4_ESM.pdf]

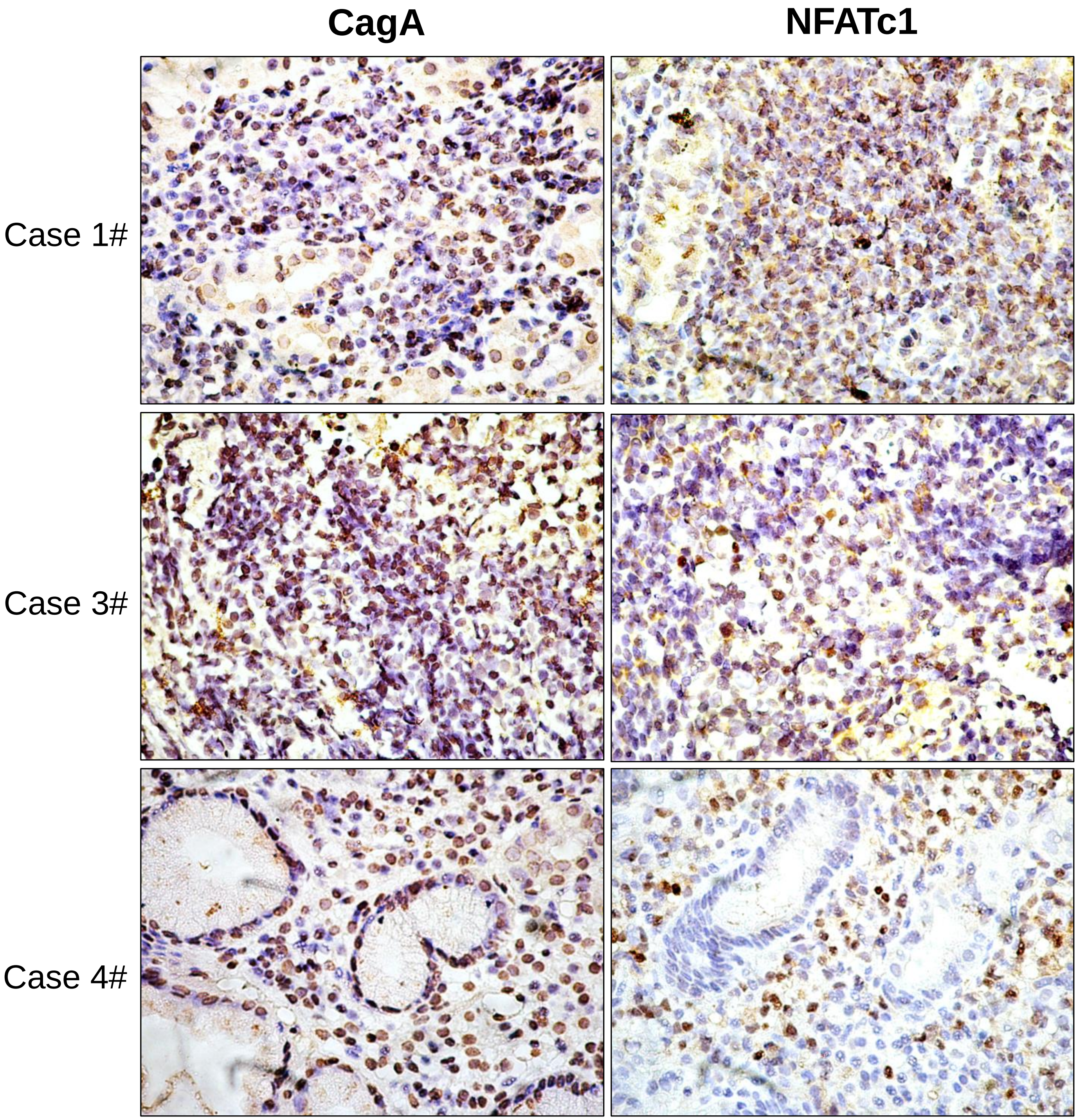

Supplementary Fig. S4. Examples of expression patterns of CagA and NFATc1 in tumor cells of HPE-responsive gastric MALT lymphoma (A) CagA expression was found in the tumor cells of gastric mucosa or submucosa in three sample cases of HPE-responsive gastric MALT lymphoma (case 1#, case 3#, and case 4#) (all images ×400) (B) Nuclear NFATc1 expression was found in the same cases of tumor cells of gastric mucosa or submucosa. (case 1#, case 3#, and case 4#) (all images ×400). CagA, cytotoxin-associated gene A; NFAT, nuclear factor of activated T cells; HP, *Helicobacter pylori*; MALT, mucosa-associated lymphoid tissue.
