# Supplementary material for: Cooperative participation of CagA and NFATc1 in the pathogenesis of antibiotics-responsive gastric MALT lymphoma
Source: Cancer Cell Int. 2024 Nov 18;24:383. doi: 10.1186/s12935-024-03552-6 (PMC11575159; doi:10.1186/s12935-024-03552-6)
Supplement: Supplementary file 5 — Supplementary material 5. [file 12935_2024_3552_MOESM5_ESM.docx]

**Cooperative participation of CagA and NFATc1 in the pathogenesis of antibiotics-responsive gastric MALT lymphoma**

**Supplementary Materials and Methods**

**Immunoblotting analysis**

# Whole cell and nuclear lysates were harvested from lymphoma and AGS cells following the various experiments. Subsequent experiments, including protein extraction, sodium dodecyl sulfate (SDS)-Tris glycine polyacrylamide gel electrophoresis (PAGE), and the application of polyvinylidene difluoride (PVDF) membranes (Millipore), primary antibodies, secondary antibodies, immune complexes, detection system for visualization, and software for quantification, were performed as previously described [32]. For immunoblotting, we used primary antibodies of specific molecules as follows: CagA (A10; sc-28368; Santa Cruz Biotechnology, Santa Cruz, CA, USA), p-CagA (tyrosine-phosphorylation, p-Tyr (PY99): sc-7020, Santa Cruz Biotechnology), NFATc1 (7A6, sc-7294; Santa Cruz Biotechnology), p-NFATc1 (Ser172; Catalog Number MAB5640, Minneapolis, MN, USA), VacA (b-300, Sc-25790; Santa Cruz Biotechnology), SHP-2 (3752; Cell Signaling, Danvers, MA, USA), p-SHP-2 (Thy542; 3751; Cell signaling), ERK (p44/42 MAPK [Erk1/2]; 9102; Cell Signaling), p-ERK (p-Erk1/2 [Thr202/Tyr204][197G2]; 4377; Cell Signaling), Bcl-xL (sc-8392, Santa Cruz Biotechnology), p21 (no. 612234; BD Transduction Laboratories, San Jose, CA, USA), p27 (no. 610241; BD Transduction Laboratories), β-actin (MAB1501, Merck, CA, USA), GAPDH (sc-32233; Santa Cruz Biotechnology), and Lamin B2 (GTX109894; GeneTex, CA, USA) [16, 17, 22, 25]. All immunoblotting tests were repeated at least thrice.

**Cell proliferation assay**

MA-1 cells (4 × 10^4^) and OCI-Ly3 cells (3 × 10^4^) in each indicated condition were seeded in 96-well culture plates and cultured for 8, 24, or 48 h. Counting of cultured cells at different time periods under the indicated conditions was performed using the trypan blue exclusion technique with a hemocytometer. Cell counting was performed in triplicate and the results are presented as the mean ± standard error.

**Cell cycle analysis**

MA-1 and OCI-Ly3 cells were pretreated with nocodazole for 22 h to synchronize the cell cycle. Subsequently, the cells were washed with fresh medium and co-cultured with or without HP HM#2, and HP HM#2 and cyclosporine A (CsA) administration in a serum-free RPMI 1640 medium for 2 h. These cells were then washed with RPMI 1640 medium and cultured in fresh RPMI 1640 medium supplemented with 10% FBS for 0.5, 1, 3, 6, or 24 h. Cultured cells were collected at the indicated time points, centrifuged, and washed with PBS. Next, these cells were fixed using 70% ethanol, stored at -20 °C overnight, subsequently washed twice with PBS, and re-suspended in 1 mL of PBS (containing 20 µg/mL of propidium iodide [Sigma-Aldrich] and 0.2 mg/mL of RNase A [Sigma-Aldrich]) for 1 h at 37 °C in the dark. We analyzed the stained cells for cell cycle progression using a FACSCalibur machine, and the data were analyzed using Modfit LT flow cytometry software.

**Assessment of the t(11;18)(q21;q21) in lymphoma cells of patients with gastric MALT lymphoma**Considering that t(11;18)(q21;q21) contributes to HPE irresponsiveness (lack of CR of lymphomas after HPE) and is a specific molecular marker for HPE-irresponsive gastric MALT lymphoma [1, 3, 8, 31, 39], we assessed the presence of t(11;18)(q21;q21) in pre-treatment lymphoma biopsies of patients with gastric MALT lymphoma. We detected the presence of t(11;18)(q21;q21) using multiplex **reverse** transcription **(RT)-PCR** followed by sequencing of the BIRC3-MALT1 fusion transcripts before 2016. Subsequently, we used a commercially available probe (*BIRC3/*MALT1 dual-color, dual-fusion translocation probe; Vysis LSI/Abbott) to detect the presence of t(11;18)(q21;q21) using interphase fluorescence in situ hybridization (FISH) [40].
