# Supplementary material for: Cooperative participation of CagA and NFATc1 in the pathogenesis of antibiotics-responsive gastric MALT lymphoma
Source: Cancer Cell Int. 2024 Nov 18;24:383. doi: 10.1186/s12935-024-03552-6 (PMC11575159; doi:10.1186/s12935-024-03552-6)
Supplement: Supplementary file 6 — Supplementary material 6. Table S1. Correlation of clinicopathological features and expression of CagA and NFATc1 with tumor response to HPE therapy in gastric MALT lymphoma without t(11;18)(p21;q21). [file 12935_2024_3552_MOESM6_ESM.docx]

| **Supplementary Table S1. Correlation of clinicopathological features and expression of CagA and NFATc1 with tumor response to HPE therapy in gastric MALT lymphoma without t(11;18)(p21;q21)** | | | | |
| --- | --- | --- | --- | --- |
|  | **Tumors response to HPE** | | |  |
| **Clinicopathological**  **characteristics** | **Total number**  **(n = 81)** | **HPE-responsive**  **(n = 59)** | **HPE-irresponsive**  **(n = 22)** | ***p**** |
| Age (median, range, years) | 55.0 (20-86) | 57.0 (30-86) | 57.0 (20-76) | 0.420# |
| Gender, men/women | 36/45 | 23/36 | 13/9 | 0.105§ |
| Endoscopic features, n (%) |  |  |  | 0.029‡ |
| Gastritis-like or multiple erosion  on infiltrative mucosa | 31 (38%) | 27 (46%) | 4 (18%) |  |
| Ulceration or ulcerated mass | 35 (43%) | 23 (396%) | 12 (55%) |  |
| Erosions on giant nodular folds | 15 (19%) | 8 (156%) | 6 (27%) |  |
| Location of lymphoma(s), n (%) |  |  |  | 0.083§ |
| Proximal^a^ or ≥ 2 components | 25 (31%) | 15 (25%) | 10 (46%) |  |
| Distal^b^ | 56 (69%) | 44 (75%) | 12 (54%) |  |
| Stage |  |  |  | 0.278§ |
| IE | 62 (77%) | 47 (80%) | 15 (68%) |  |
| IIE1 | 19 (23%) | 12 (20%) | 7 (32%) |  |
| Depth of gastric wall involvement, n (%)¶ |  |  |  | 0.010§ |
| Submucosa or above | 45/76 (59%) | 38/56 (68%) | 7/20 (35%) |  |
| Muscularis propria or beyond | 31/76 (41%) | 18/56 (32%) | 13/20 (65%) |  |
| CagA expression |  |  |  | < 0.001§ |
| Positive | 53 (65%) | 47 (81%) | 6 (27%) |  |
| Negative | 28 (35%) | 12 (19%) | 16 (73%) |  |
| NFATc1 |  |  |  | < 0.001§ |
| Positive | 50 (55%) | 43 (73%) | 7 (32%) |  |
| Negative | 31 (45%) | 16 (27%) | 15 (68%) |  |
| Abbreviation; HPE, *Helicobacter pylori* eradication therapy; MALT, mucosa-associated lymphoid tissue  *p**: comparison of discrete variables between HPE-responsive cases and HPE-irresponsive cases  #*p* values (two-sided) were calculated using the Student’s t-test  §*p* values (two-sided) were calculated using Chi-square test or Fisher’s exact test.  ‡*p* values (two-sided) were calculated using one-way analysis of variance.  Proximal^a^: Middle body, upper body, fundus, or cardia. Distal^b^: Antrum, angle, or lower body.  ¶ Gastric wall involvement was evaluated by endoscopic ultrasonography in 76 patients. | | | | |
