# Supplementary material for: Cooperative participation of CagA and NFATc1 in the pathogenesis of antibiotics-responsive gastric MALT lymphoma
Source: Cancer Cell Int. 2024 Nov 18;24:383. doi: 10.1186/s12935-024-03552-6 (PMC11575159; doi:10.1186/s12935-024-03552-6)
Supplement: Supplementary file 7 — Supplementary material 7. Table S2. Clinicopathological features and NFATc1 expression in patients with stage IE/IIE1 gastric MALT lymphoma without t(11;18)(p21;q21) who received first-line HPE therapy. [file 12935_2024_3552_MOESM7_ESM.docx]

| **Supplementary Table S2. Clinicopathological features and NFATc1 expression in patients with stage IE/IIE1 gastric MALT lymphoma without t(11;18)(p21;q21) who received first-line HPE therapy** | | | | |
| --- | --- | --- | --- | --- |
|  | **NFATc1 expression** | | |  |
| **Clinicopathological characteristics** | **Total number**  **(n = 81)** | **Positive**  **(n = 50)** | **Negative**  **(n = 31)** | ***p**** |
| Age (median, range, years) | 55.0 (20-86) | 57.0 (30-83) | 54.0 (20-86) | 0.683# |
| Sex, men/women | 36/45 | 21/29 | 15/16 | 0.574§ |
| Endoscopic features, n (%) |  |  |  | 0.561‡ |
| Gastritis-like or multiple erosion  on infiltrative mucosa | 31 (38%) | 19 (39%) | 12 (39%) |  |
| Ulceration or ulcerated mass | 35 (43%) | 20 (40%) | 15 (48%) |  |
| Erosions on giant nodular folds | 15 (19%) | 11 (22%) | 4 (13%) |  |
| Location of tumor (s), n (%) |  |  |  | 0.831§ |
| Proximal^a^ or ≥ 2 components | 25 (31%) | 15 (30%) | 10 (32%) |  |
| Distal^b^ | 56 (69%) | 35 (70%) | 21 (68%) |  |
| Stage |  |  |  | 0.493§ |
| IE | 62 (77%) | 37 (74%) | 25 (81%) |  |
| IIE1 | 19 (23%) | 13 (26%) | 6 (19%) |  |
| Depth of gastric wall involvement, n (%)¶ |  |  |  | 0.297§ |
| Submucosa or above | 45/76 (59%) | 30/47 (64%) | 15/29 (52%) |  |
| Muscularis propria or beyond | 31/76 (41%) | 17/47 (36%) | 14/29 (48%) |  |
| CagA expression |  |  |  | < 0.001§ |
| Positive | 53 (65%) | 40 (80%) | 13 (42%) |  |
| Negative | 28 (35%) | 10 (20%) | 18 (58%) |  |
| Abbreviation; HPE, *Helicobacter pylori* eradication therapy; MALT, mucosa-associated lymphoid tissue  *p**: comparison of discrete variables between NFATc1-positive cases and NFATc1-negative cases  #*p* values (two-sided) were calculated using the Student’s t-test  §*p* values (two-sided) were calculated using Chi-square test or Fisher’s exact test.  ‡*p* values (two-sided) were calculated using one-way analysis of variance.  Proximal^a^: Middle body, upper body, fundus, or cardia. Distal^b^: Antrum, angle, or lower body.  ¶ Gastric wall involvement was evaluated by endoscopic ultrasonography in 76 patients. | | | | |
