# Supplementary material for: Cooperative participation of CagA and NFATc1 in the pathogenesis of antibiotics-responsive gastric MALT lymphoma
Source: Cancer Cell Int. 2024 Nov 18;24:383. doi: 10.1186/s12935-024-03552-6 (PMC11575159; doi:10.1186/s12935-024-03552-6)
Supplement: Supplementary file 8 — Supplementary material 8. Table S3. Positive predictive values of CagA and NFATc1 for HPE responsiveness in stage IE/IIE1 gastric MALT lymphoma without t(11;18)(p21;q21). [file 12935_2024_3552_MOESM8_ESM.docx]

**Supplementary Table S3. Positive predictive values of CagA and NFATc1 for HPE responsiveness in stage IE/IIE1 gastric MALT lymphoma without t(11;18)(p21;q21)**

| Expression of CagA and NFATc1 molecules  in tumor tissue | Number of patients with gastric MALT lymphoma who received first-line HPE | | |
| --- | --- | --- | --- |
|  | HPE-responsive  (n = 59) | HPE-irresponsive  (n = 22) | |
| CagA positive | 47 | 6 |  |
| CagA negative | 12 | 16 |  |
| PPV* for CagA expression = 47 of 53 (88.7%) |  |  |  |
| Specificity** for CagA expression = 16 of 22 (72.7%) |  |  |  |
| NFATc1 positive | 43 | 7 |  |
| NFATc1 negative | 16 | 15 |  |
| PPV* for NFATc1 expression = 43 of 50 (86.0%) |  |  |  |
| Specificity** for NFATc1 expression = 15 of 22 (68.2%) |  |  |  |
| CagA and NFATc1 are all positive |  |  |  |
| Yes | 38 | 3 |  |
| No | 21 | 19 |  |
| PPV¶ for combined CagA and NFATc1 = 38 of 41 (92.7%) |  |  |  |
| Specificity§ for combined CagA and NFATc1 = 19 of 22 (86.4%) |  |  |  |

Abbreviation: MALT. Mucosa-associated lymphoid tissue; HPE, *Helicobacter pylori* eradication

*Positive predictive value (PPV) = Number of HPE-responsive cases who had CagA expression or nuclear NFATc1 localization/Total positive cases for respective CagA expression or nuclear NFATc1 localization.

**Specificity = Number of HPE-irresponsive cases who had no CagA expression or nuclear NFATc1 localization/Total HPE-irresponsive cases.

¶PPV = Number of HPE-responsive cases who had all CagA and nuclear NFATc1 localization/Total cases expressing all CagA and nuclear NFATc1 localization.

§Specificity = Number of HPE-irresponsive cases who did not simultaneously express CagA and nuclear NFATc1 localization in tumors/Total HPE-irresponsive cases.
